# Curcumin‐loaded cockle shell‐derived calcium carbonate nanoparticles ameliorates lead‐induced neurotoxicity in rats via attenuation of oxidative stress

**DOI:** 10.1002/fsn3.3096

**Published:** 2022-10-30

**Authors:** Maryam Muhammad Mailafiya, Kabeer Abubakar, Samaila Musa Chiroma, Abubakar Danmaigoro, Tawfiq Y. T. Zyoud, Ezamin Bin Abdul Rahim, Mohamad Aris Mohd Moklas, Zuki Abu Bakar Zakaria

**Affiliations:** ^1^ Department of Human Anatomy, Faculty of Medicine and Health Sciences University Putra Malaysia Serdang Malaysia; ^2^ Department of Human Anatomy College of Medicine Federal University Lafia Lafia Nigeria; ^3^ Department of Human Anatomy, Faculty of Basic Medical Sciences University of Maiduguri Maiduguri Nigeria; ^4^ Department of Veterinary Anatomy, Faculty of Veterinary Medicine Usman Danfodiyo University Sokoto Nigeria; ^5^ Department of Radiology, Faculty of Medicine and Health Sciences University Putra Malaysia Serdang Malaysia; ^6^ Department of Preclinical Sciences Faculty of Veterinary Medicine University Putra Malaysia Serdang Malaysia

**Keywords:** cockle shell, curcumin, drug delivery, lead, nanoparticles, neurotoxicity, oxidative stress

## Abstract

A substantial global health burden is associated with neurotoxicity caused by lead (Pb) exposure and the common mechanism of this toxicity is mainly via oxidative damage. Curcumin has remarkable pharmacological activities but remains clinically constrained due to its poor bioavailability when orally administered. Currently, cockle shell‐derived calcium carbonate nanoparticle (CSCaCO_3_NP) is gaining more acceptance in nanomedicine as a nanocarrier to various therapeutics. This study aimed at investigating the ameliorative effect of curcumin‐loaded CSCaCO_3_NP (Cur‐CSCaCO_3_NP) on lead‐induced neurotoxicity in rats. A total of 36 male Sprague–Dawley rats were randomly assigned into five groups. Each group consists of 6 rats apart from the control group which consists of 12 rats. During the 4 weeks induction phase, all rats received a flat dose of 50 mg/kg of lead while the control group received normal saline. The treatment phase lasted for 4 weeks, and all rats received various doses of treatments as follows: group C (Cur 100) received 100 mg/kg of curcumin, group D (Cur‐CSCaCO_3_NP 50) received 50 mg/kg of Cur‐CSCaCO_3_NP, and group E (Cur‐CSCaCO_3_NP 100) received 100 mg/kg of Cur‐CSCaCO_3_NP. The motor function test was carried out using the horizontal bar method. The cerebral and cerebellar oxidative biomarker levels were estimated using ELISA and enzyme assay kits. Lead‐administered rats revealed a significant decrease in motor scores and SOD activities with a resultant increase in MDA levels. Furthermore, marked cellular death of the cerebral and cerebellar cortex was observed. Conversely, treatment with Cur‐CSCaCO_3_NP demonstrated enhanced ameliorative effects when compared with free curcumin treatment by significantly reversing the aforementioned alterations caused by lead. Thus, CSCaCO_3_NP enhanced the efficacy of curcumin by ameliorating the lead‐induced neurotoxicity via enhanced attenuation of oxidative stress.

## Graphical abstract

Treatment effect of Nanocurcumin

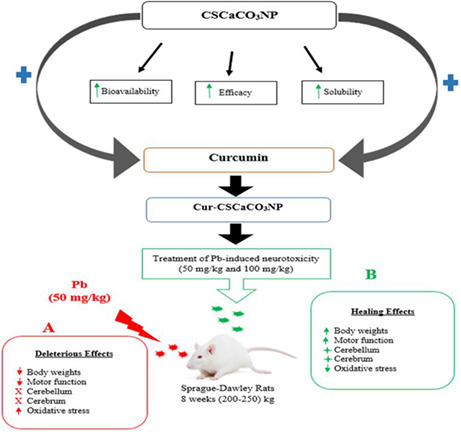

## 1 INTRODUCTION

Lead (Pb) is a ubiquitous environmental toxic metal that is used in agriculture and modern industries, which consequently causes numerous harmful health effects to man and it is now an important public health burden (Patrick, 2006a). The continuous usage of lead owing to its beneficial physicochemical properties yet harmful to health is from antiquity to modern days (Ansar et al., 2019; McQuirter et al., 2013). Consequently, continuous human exposure to lead is becoming inevitable leading to a significant global challenge (Carrington et al., 2019; Ming et al., 1997). Several health consequences in adults are linked to occupational exposure to lead which remained a major source of lead toxicity (Bhattacharjee et al., 2018). Lead can interrupt normal biological function by causing inflammations and oxidative stress via several pathways leading to cell degeneration and death (Lakshmi et al., 2013; Mason et al., 2014). Neurotoxins such as lead produces an adverse effect on the nervous system resulting in several neuropathological and neurological disorders (Wani et al., 2015). Lead‐induced neurotoxicity and neurological disorders are characterized by cognitive deficit, impaired motor function, attention deficit, dullness, low IQ, hyperactivity, and antisocial problems among others (Vlasak et al., 2019). Preceding studies on humans and experimental animals have documented several lead‐induced neurotoxic insults. For example, a significant decrease in motor scores and SOD activity with a resultant increase in MDA with evidence of lead concentration, which resulted in marked histological alterations in the cerebellar cortex of rats induced with 50 mg/kg of lead, were documented (Abubakar, Muhammad Mailafiya, et al., 2019); also, a study has shown that rats exposed to lead at 7.5 mg/kg body weight for 14 days resulted in significant oxidative alterations and histological degeneration in the rats' cerebral cortex and blood–brain barrier (Singh et al., 2017), documented studies reported encephalopathy as a direct consequence of lead exposure which is characterized by dullness, irritability, headache, attention deficit, loss of memory, hearing loss, etc. in children exposed to lead poisoning (Paul & Gupta, 2018), and another study linked the lead exposure to be the major cause of peripheral nervous system dysfunction in adult while the central nervous system is more prominently affected in children (Bellinger et al., 2018; Bose‐O'Reilly et al., 2017; Plumlee et al., 2013).

Noteworthy, in the absence of consistent standard neurotherapeutic drugs in allopathic medicine, herb extracts display therapeutic functions in the treatment of many lead‐induced neurotoxicity and other related organ toxicity (Hewlings & Kalman, 2017; Shaikh et al., 2009). Nevertheless, many of these available herbs are insoluble, which limits their absorption and subsequent bioavailability (Marslin et al., 2018; Paul & Gupta, 2018; Sharma et al., 2005). Among the insoluble herbs, curcumin possessed an old documented medicinal history that mirrored the current field of nanomedicine, drawing numerous attention of researchers due to its wide safety margin and health benefits such as antioxidant, anti‐inflammatory, and neurotherapeutic activities (Chirio et al., 2019; Mofazzal Jahromi et al., 2014). In spite of all the commendable curative properties of curcumin, the major drawback of poor bioavailability due to poor aqueous solubility, poor absorption from the intestine, rapid metabolism in the liver, and high degree of elimination in the bile has constrained its clinical applications (Priyadarsini, 2014; Yadav et al., 2012). Hence, searching for a safe and potential delivery system that will overcome such limitations to ensure safe delivery within a biological system thereby enhancing curcumin therapeutic efficacy has become the most fascinating and desired area of research in nanotechnology (Basniwal et al., 2014).

Cockle shells from a natural marine source have recently been used as a nanocarrier for the delivery of various therapeutic agents for chemotherapy and antibacterial purposes (Danmaigoro et al., 2017; Hammadi et al., 2017; Isa et al., 2016). This natural biogenic material is a strong source of abundant calcium carbonate existing in aragonite polymorphic form (Hoque et al., 2014; Mailafiya, Abubakar, Danmaigoro, et al., 2019). Its outstanding potential ability to safely deliver several anticancer and antibacterial agent was demonstrated in previous literatures (Fu et al., 2017; Hamidu et al., 2019; Isa et al., 2016). To date, no study to the best of our knowledge have yet documented cockle shell‐derived calcium carbonate nanoparticles (CSCaCO_3_NP) as effective delivery of antioxidant such as curcumin in vivo.

Exposure to metals such as lead has been reported to be one of the leading causes of cerebral and cerebellar toxicity (Sidhu & Nehru, 2004). Cerebrum is the largest part of the brain responsible for superior brain functions such as motor movement, emotions, learning, and recognition, while the cerebellum is the major structure of the hindbrain responsible for motor coordination and balance (Lazarus et al., 2018; Mahmoud & Sayed, 2016). Both cerebellum and cerebrum are delicate structures that are vulnerable to intoxication resulting in a deficit of cognitive abilities and impaired motor coordination and balance (Bhattacharjee et al., 2018; Patrick, 2006b). A previous study reported the common direct culprit of environmental lead exposure to be via ingestion, particularly in drinking water. (Bhattacharjee et al., 2018) Furthermore, lead exposure even at a low level resulted in several pathological conditions with a great impact on the nervous system of the populations exposed (Flora et al., 2012; Husain, 2015). Thus, regardless of the higher amount of lead exposure, cumulative dose of lead and vulnerability of the individual are strongly linked to health consequences (Bose‐O'Reilly et al., 2017; Kim et al., 2014). Hence, in this study, the choice of oral administration of lead at a dose of 50 mg/kg was adopted in order to mimic the environmental exposure of lead to organisms.

The dose of free curcumin at 100 mg/kg revealed non‐toxic effects in rats; in fact, a higher dose of curcumin showed no sign of toxicity, indicating its wide safety margin (Sarada et al., 2015; Zhang et al., 2018). Furthermore, the toxicity evaluation of CSCaCO_3_NP in both rats and dogs indicated that CSCaCO_3_NP have a wide safety margin to the biological system in vivo (Danmaigoro et al., 2018; Jaji, Zakaria, et al., 2017). In addition, several in vitro studies reported the great safety and biocompatibility effect of CSCaCO_3_NP on various cell lines (Fu et al., 2017; Hamidu et al., 2019; Kamba et al., 2014; Mailafiya, Moklas, et al., 2019).

Previous studies have emphasized the targeted effects of different nanoparticles for curcumin's delivery for the treatment of various heavy metals‐induced neurodegenerative diseases (Kakkar & Kaur, 2011; Sandhir et al., 2014). However, the current research may be an added advantage because it stressed not only on the targeted effect mechanism of CSCaCO_3_NP but also the ability of the nanoparticle to enhance the therapeutic effect of curcumin. Therefore, the study generally aimed at evaluating the ameliorative effect of curcumin‐loaded cockle shell‐derived calcium carbonate nanoparticles (Cur‐CSCaCO_3_NP) on lead‐induced neurotoxicity in rats via behavioral, biochemical, histological, and histochemical assessments.

## 2 MATERIAL AND METHODS

### 2.1 Chemical, reagents, and kits

Cockle shell was obtained from Malaysian local wet market. Lead acetate (99%), curcumin, and rat chow were purchased from Sigma‐Aldrich (St. Louis, MO, USA). Toluidine blue stain was purchased from Agar Scientific (Agar Scientific Ltd, UK). Fixatives and H&E stain were purchased from Sigma Aldrich, St. Louis Co., United State and normal saline was obtained from Apical Scientific Sdn, Bhd Malaysia. Kits include ELISA kit (Elabscience biotechnology Inc.), Pierce™ BCA protein assay kit (Thermo Fisher Scientific, Carlsbad, CA, USA), and superoxide dismutase (SOD) assay kit (E‐BC‐K020, Elabscience Biotechnology Inc.). All other reagents and chemicals used were of high analytical grade quality and higher purity.

### 2.2 Animals

Thirty‐six (36) healthy, adult, male Sprague–Dawley rats aged 8 weeks with an average body weight ranging between 200 and 250 g were used in this study. The rats were obtained from the Animal Breeding Unit, Faculty of Veterinary Medicine, Universiti Putra Malaysia. The rats were kept in plastic cages and maintained under the same laboratory conditions (temperature of 25°C ± 2°C and 12 h light:12 dark cycles) for 1 week of acclimatization. All rats had free access to rat chow and water ad libitum during the study period. The animal management and care procedures were performed according to the Organization for Economic Cooperation and Development (OECD) recommended guidelines. The experiment was conducted with the approval of the Animal Care Committee (IACUC) of the Universiti Putra Malaysia (UPM/IACUC/AUP‐R038/2018), approved in September 2018.

### 2.3 Synthesis of CSCaCO_3_NP and Cur‐CSCaCO_3_NP

The preparation, synthesis, loading processes, and physicochemical characterizations of CSCaCO_3_NP and Cur‐CSCaCO_3_NP as well as the in vitro kinetic release were previously described in the work of Mailafiya, Abubakar, Danmaigoro, and Musa (2019) Noteworthily, based on the protocol and procedure of the author's previous study, the best formulation of Cur‐CSCaCO_3_NP that gives the best loading content and good encapsulation efficiency was selected for curcumin delivery.

### 2.4 Experimental design

**FIGURE 1:**
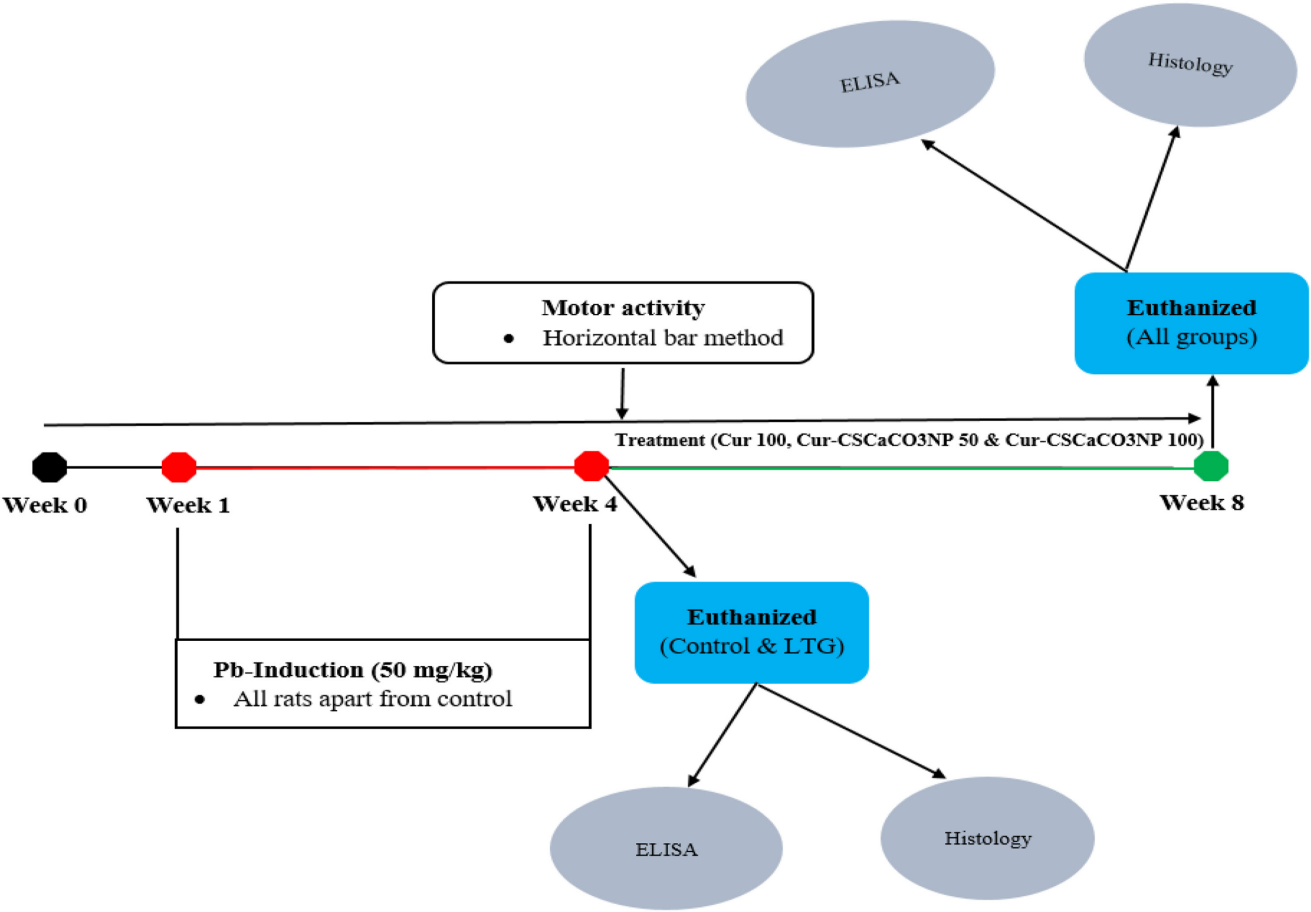
Schematic diagram of the experimental design showing complete periodic activities of the experimental rats. *Note:* Lead‐treated groups (LTG), curcumin 100 mg/kg (cur 100), curcumin‐loaded cockle shell‐derived calcium carbonate nanoparticles at the dose of 50 and 100 mg/kg, respectively (Cur‐CSCaCO_3_NP 50 and 100), enzyme‐linked immunosorbent assay (ELISA), lead (lead).

Following 1 week of acclimatization, the rats were randomly assigned into five groups (A, B, C, D, and E), comprising six rats each; except for control group A, which consists of 12 rats. The groups were as follows: A: the control group (normal saline), B: lead‐treated group (LTG), and three treatment groups (C: lead and free curcumin, 100 mg/kg; D: lead and Cur‐CSCaCO_3_NP, 50 mg/kg; and E: lead and Cur‐CSCaCO_3_NP, 100 mg/kg). A motor activity test was conducted (from week 0 to week 8). The experimental design consists of the induction phase that involved the 4 weeks of oral administration of lead at a flat dose of 50 mg/kg to all the rat's groups except the control group (A). The method of lead induction was in accordance with the procedures of Owolabi et al. (2012) and Ayuba and Ekanem (2017) and it took place three times a week. The overall experimental administration method was in accordance with the work of Sankar et al. (2013) with slight modification. At the end of the first phase (week 4), six rats from normal control group A and the whole of group B were euthanized to confirm the lead toxicity. The second phase of the experiment commenced with the oral treatment of the rats with free curcumin at a dose of 100 mg/kg for group C and Cur‐CSCaCO_3_NP at a dose of 50 and 100 mg/kg for groups D and E, respectively (three times in a week). The induction phase and treatment phase lasted for 4 weeks each. The overall experiment lasted for 8 weeks (Figure 1).

### 2.5 Dose preparation of lead and Cur‐CSCaCO_3_NP

The lead solution was prepared by dissolving 1 g of lead acetate in 50 ml of deionized water to form a stock solution of 20 mg/ml of lead acetate concentration. Each rat in all the groups with the exception of the rats from control group A was given a dose of 50 mg/kg three times based on their body weight (Ayuba & Ekanem, 2017; Owolabi et al., 2012; Sidhu & Nehru, 2004).

The Cur‐CSCaCO_3_NP was weighed to get the exact number of milligrams per kg needed for the rats (i.e., 100 and 50 mg/kg) from which a stock solution was made by dissolving it in 50 ml of deionized water. Each rat (in groups D and E) was given a dose of 50 and 100 mg/kg three times a week, respectively, based on the body weight.

### 2.6 Weekly body weight measurement and physical observations

During the period of the experiment, normal physical activities of the rats were observed: daily water intake, voluntary feed intake, fecal output, weight loss, and gain were observed. Starting from week 0 to week 8, weight gain and weight loss were recorded at 1‐week intervals for each rat. This was done to monitor the trend of body weight throughout the period of the experiment. The recorded body weights within the study period were subjected to statistical analysis using two‐way ANOVA.

### 2.7 Motor activity test

To monitor the trend of the rat's ability of motor coordination, their forelimb grip balance was tested starting from week 0 to week 8 using a horizontal bar method. The rats were transported into the training room 1 h before the start of the experiment for the rats to adjust to their new environment. The entire motor activity test was done between 9 am and 1 pm.

### 2.8 Horizontal bar method (HBM)

The method measures forelimb strength and coordination. The rat's ability to grip the bar using their phalanges was assessed weekly. This method involves the use of 38‐cm‐long and 2‐mm‐diameter metal bar, suspended horizontally above 49 cm height with end‐to‐end supports of a laboratory clamp, and a padded surface to ensure rat's soft landing when falling. Each rat was held by the tail, carefully placed at the central point of the metal bar, and allowed its forepaws to grasp the bar, and the tail was released immediately after grasping at the same time the stopwatch was started to measure the time. The translation of the time into scores in this study was done in accordance with the intense description by the previous work Deacon (2013). The scoring procedure is shown in Table 1.

**TABLE 1:** Horizontal bar method scoring system (Deacon, 2013)

Falls	Time (seconds)	Scores
Falling between	1–5	1
Falling between	6–10	2
Falling between	11–20	3
Falling between	21–30	4
Falling after	30	5
Without falling	‐	5

*Note*: If the experimental animal grasps the bar properly and moves from end to end of the bar without falling, then a maximum score (5) was allotted. All rats underwent the test in three different attempts with brief resting intervals to obtain the best score and prevent error.

The horizontal bar method in this study was used to assess the effect of Cur‐CSCaCO_3_NP on the motor coordination initiated by the cerebrum of lead‐induced rats. However, to avoid possible confounding factors that may arise when investigating the effect of Cur‐CSCaCO_3_NP in the brain of lead‐induced Sprague–Dawley rats for the first time, this research confirmed the use of male instead of female gender. This is because female cyclical hormonal changes usually affect their mood swings, thus, sex hormones such as prolactin, progesterone, and estrogen may arbitrarily influence feeding habit, emotion, motor behavior, and cognitive function during the experiments as stated in previous literatures (Chiroma et al., 2019; Frye, 2010).

### 2.9 Sample collection

At the end of the experimental period (8 weeks), all the rats were euthanized, and the brain was harvested, washed thrice in ice‐cold saline, and weighed. The brain tissues were separated into two; one portion was stored at −80°C for SOD and ELISA assays and the other portion was preserved in 10% buffered formalin for histological and histochemical analyses. The brain tissue stored at −8°C for MDA and SOD assays was allowed to thaw. Cerebellum and cerebrum were isolated and then homogenized with ice‐cold phosphate‐buffered saline (PBS) (0.01 M, pH = 7.4) in a volume of 20 times the weight of the tissue to prepare 10% cerebral and cerebellar homogenates at the ratio of 9:1. The homogenates were centrifuged at 5000 × *g* for 5 min at 4°C and the final aliquot of the supernatant was separated and kept at −80°C.

### 2.10 Protein estimation

The total protein concentration of the cerebrum and cerebral tissues was measured using the bicinchoninic acid assay (BCA assay). The standard used was bovine serum albumin (BSA) (2 mg/ml) with a working range between 125 and 2000 μg/ml (Appendix S1).

### 2.11 ELISA and SOD activity analyses

Malondialdehyde (MDA) level was detected from the rats' cerebellum and cerebrum homogenates base on the simple principle of competitive ELISA using the MDA ELISA kit (E‐EL‐006, Elabscience). MDA level was assayed by monitoring the competitive reaction of the MDA in the tissue samples with the fixed amount of MDA on the precoated microtiter plate surface. A standard working solution was set up for each sample well and 50 μl of the sample was added to each well separately. Followed by the immediate addition of biotinylated detection Ab working solution to each well, sealed and incubated for 45 min at 37°C. Next, the solutions were aspirated, and 350 μl of buffer was added to each well and soaked for 2 min then aspirated again three times before the addition of 100 μl of HRP conjugate working solution to each well, and incubated for 30 min at 37°C. Furthermore, the incubated solution was decanted and washed with buffer solution five times before the addition of 90 μl of substrate reagent to each well and incubated for 15 min at 37°C. Finally, 50 μl of stop solution was added to each well and the absorbance was measured using a microplate reader at a wavelength of 450 nm. The standard calibration curve was set up for this assay (Appendix S2) and the results were expressed as ng/ml.

The cerebellum and cerebrum homogenates were further analyzed to check for the SOD activities using the method of colorimetric analysis of WST‐1 principle strictly based on the kit's instructions. The reaction mixture consisting of 20 μl of tissue homogenates, 20 μl of enzyme working solution, and 200 μl of substrate application solution was fully mixed and incubated at 37°C for 20 min. The absorbance was taken at a wavelength of 450 nm and the results were expressed in U/mg prot.

### 2.12 Histopathological analysis

#### 2.12.1 Hematoxylin and eosin (H&E)

The fixed tissues were processed for histological evaluation as earlier described by Danmaigoro et al. (2018) Briefly, the brain tissues were trimmed and dehydrated in ascending concentration of alcohol, cleared in xylene, and further embedded in paraffin wax. Furthermore, the tissue was trimmed and sectioned to approximately 5 μm thick in size. The sectioned brain tissues were stained using the techniques for standard Harris's hematoxylin and eosin for normal histology and histopathological studies and examined under the light microscope. The degree of tissue injury, necrosis, and inflammatory responses were analyzed.

### 2.13 Histopathological tissue scoring system

For scoring, through a guide of an independent histologist, a minimum of three sections of each tissue were taken for evaluation in five rats per group (*n* = 5). Quantification of the non‐viable (affected) pyramidal cells and Purkinje cells was done in 10 different nonoverlapping fields of cerebral cortex and cerebellum tissue sections under 400× magnification and the average mean for each group was analyzed. This was done manually with the aid of an image analyzer in a blinded manner by independent pathologists. The overall methods of the tissue scoring system were in accordance with previous literature (Partadiredja et al., 2013).

### 2.14 Histochemical analysis

The sectioned ribbon brain tissues (cerebellum and cerebrum) were stained using toluidine blue as a special stain. The following reagents were used to prepare the stain; colophonium (resin) 10 g, 95% alcohol 100 ml, toluidine blue 0.1 g, Distilled water 100 ml, and 10% solution of aniline in 95% alcohol. The section tissues were totally immersed in xylene, absolute alcohol, and 95% alcohol. After which they were dipped in alcoholic colophonium solution for 3–5 min and rinsed in two changes of 95% alcohol (3 min each), then stained with toluidine blue (30 s), followed by differentiation in aniline–alcohol and cleared in xylene (two changes) again and, finally, mounted in synthetic resin.

### 2.15 Statistical analysis

All analyses were conducted using GraphPad Prism (GraphPad Prism software, Inc, Version 6.01, San Diego, California, USA) and SPSS. Differences in *p* values <0.05 were statistically significant for the purpose of comparison. The data obtained were presented as mean ± standard error of the mean (SEM). Data obtained from the horizontal bar method (HBM) and weekly body weight (WBW) were analyzed using repeated measures followed by Tukey's post hoc test. The data obtained from the effect of lead on various parameters were conducted using Student's unpaired samples *t*‐test while the data obtained from histology, ELISA, and SOD analysis were all analyzed by one‐way ANOVA followed by Tukey's post hoc test.

## 3 RESULTS

### 3.1 Physical observations

At the early weeks of lead induction, the rats showed no pronounced physical evidence of toxicity. Subsequently, decreased feed and water intake with minimal gross evidence of toxicity (i.e., rough fur and slight body weakness) were observed among all the lead‐treated groups of rats in the subsequent weeks of lead induction. However, treatments with Cur‐CSCaCO_3_NP remarkably improved the eating habit of the rats and reduced their body weakness.

### 3.2 Effect of lead on the motor functions of rats

**FIGURE 2:**
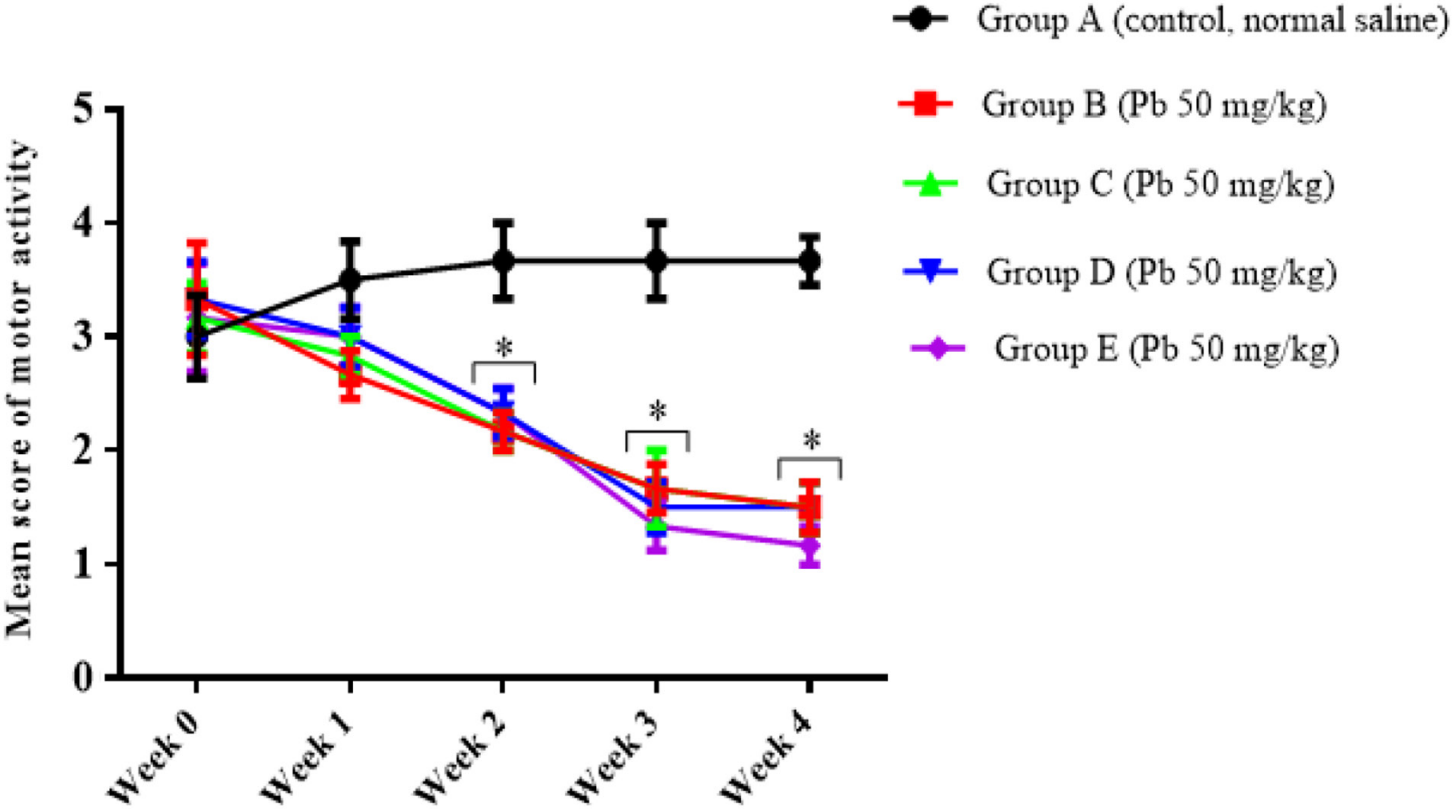
Effect of lead on the motor score of rats after 4 weeks of induction. Values were presented as mean ± SEM, *n* = 6. *p < 0.05 versus control group.

Statistically significant interactions between the effect of lead induction and the weeks of induction [*F* (16, 100) = 3.414, *p* = 0.0001] in the motor activity scores of the rats were observed after lead induction. Tukey's multiple‐comparison test revealed a statistically significant decrease (*p* < 0.05) in the motor score of the lead‐induced rats for the ability to maintain a forelimb grip balance on week 2, 3, and 4 by groups B, C, D, and E when compared to the control group of rats (Figure 2).

### 3.3 Ameliorative effects of Cur‐CSCaCO_3_NP on the motor score of rat motor functions

**FIGURE 3:**
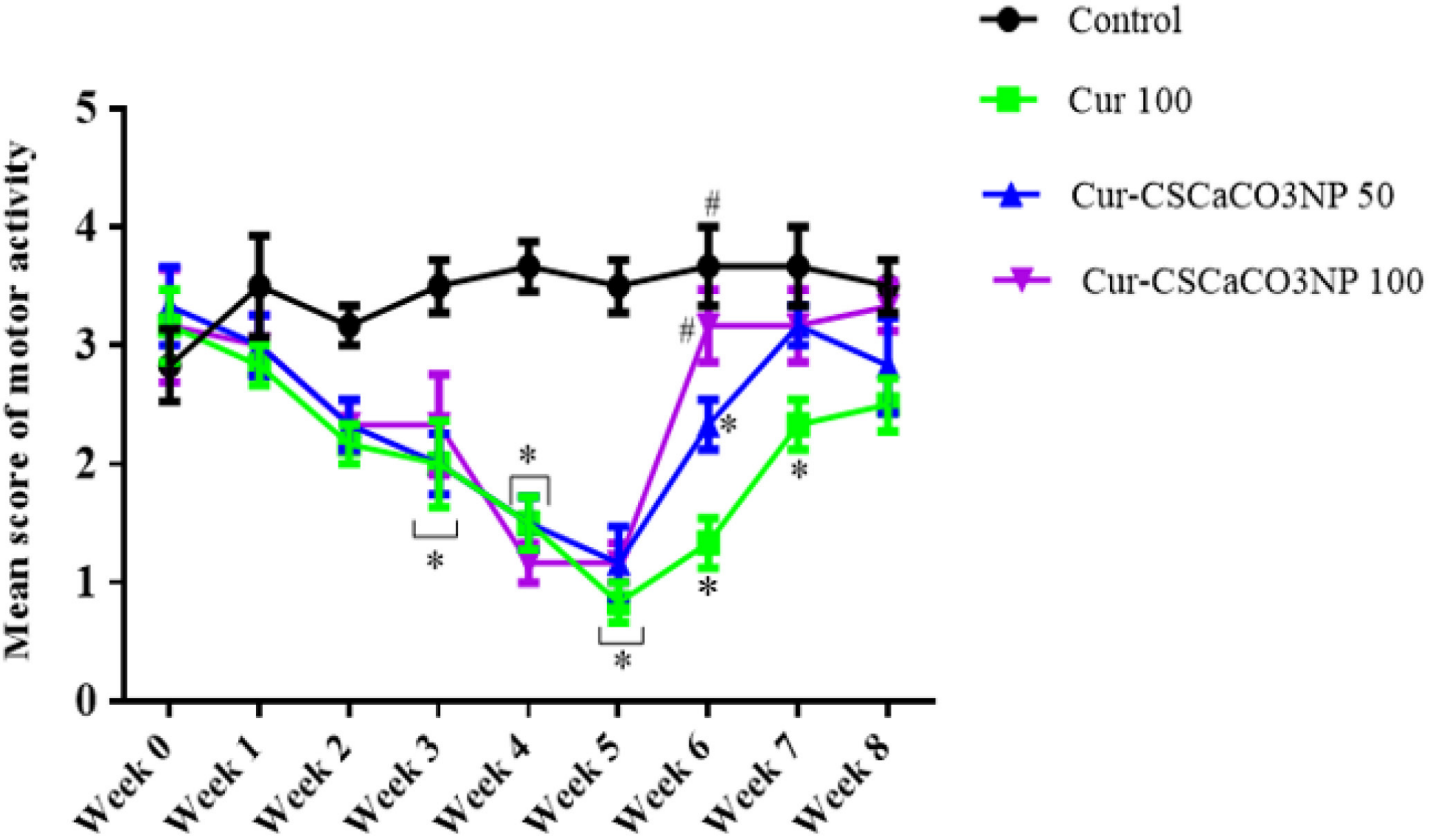
Effect of cur‐CSCaCO_3_NP and curcumin on the motor score of rats exposed to lead. Values were presented as mean ± SEM, *n* = 6. *p < 0.05 versus control, #*p* < 0.05 versus Cur 100.

Repeated measures showed a statistically significant interaction between the effect of treatment and the weeks of treatment [*F* (24, 160) = 3.780, *p* < 0.0001] in the motor activities score of the rats. Tukey's post hoc test showed a statistically significant decrease (*p* < 0.05) in the motor score of the rats for their ability to maintain a forelimb grip balance on weeks 3, 4, and 5 by the Cur 100, Cur‐CSCaCO_3_NP 50, and Cur‐CSCaCO_3_NP 100 groups when compared to the control group of rats. Subsequently, a similar trend was also observed on week 6 of the test where a statistically significant decrease (*p* < 0.05) in the motor score of the rats for their ability to maintain a forelimb grip balance by rats of Cur 100 and Cur‐CSCaCO_3_NP 50 groups when compared to the control group. Conversely, a statistically significant increase (*p* < 0.05) was observed in the motor score of the rats on week 6 by the control and Cur‐CSCaCO_3_NP 100 groups when compared to the Cur 100 group of rats. Furthermore, a statistically significant decrease was observed (*p* = 0.0367) in the motor score of the rats for their ability to maintain a forelimb grip balance on week 7 by the Cur 100 when compared to the control group of rats. No statistically significant differences were observed in all the groups when compared to the control group on week 8 as shown in Figure 3.

### 3.4 Effect of lead on body weight of rats

**FIGURE 4:**
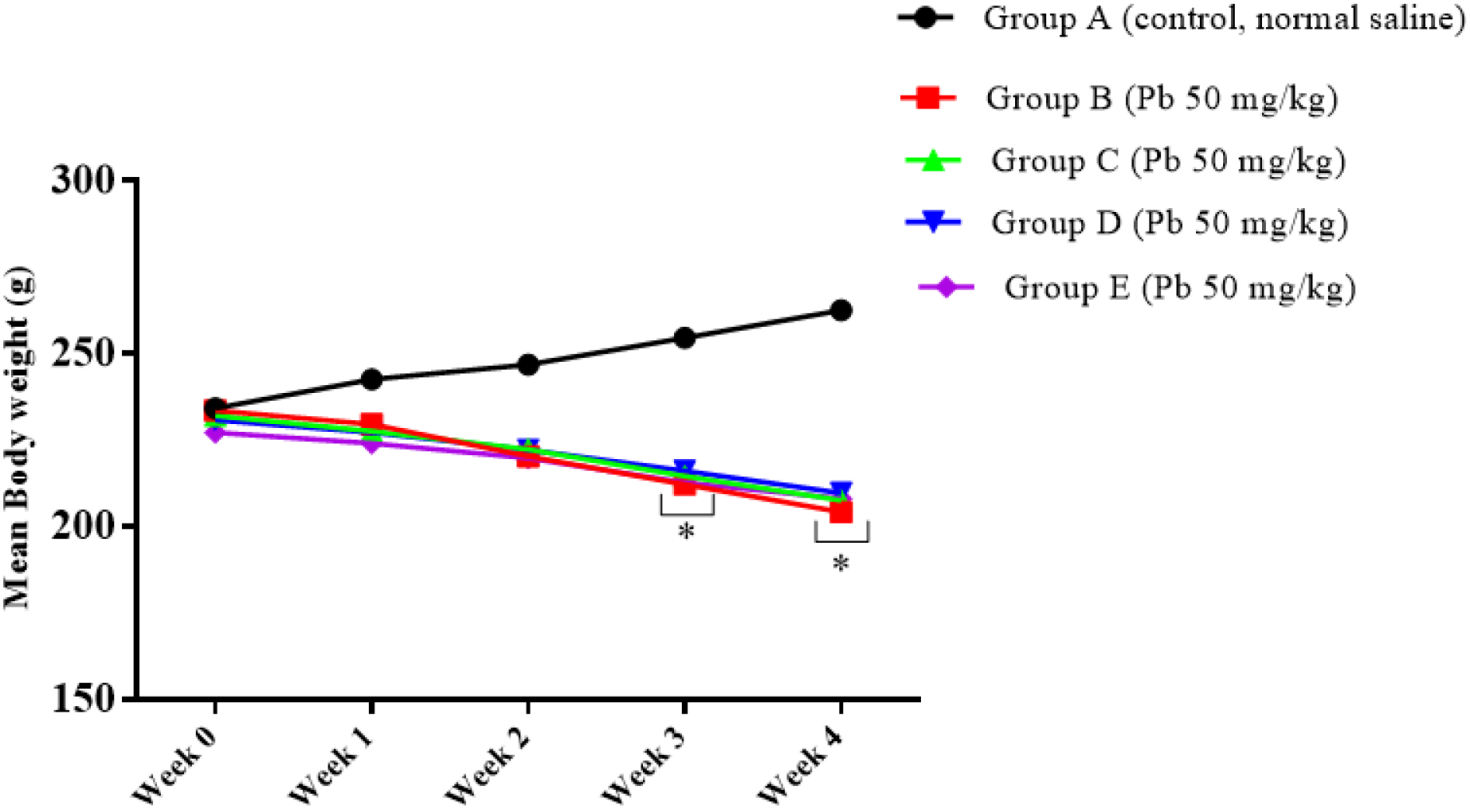
Effect of lead on the rats' body weight after 4 weeks of induction. Values were presented as mean ± SEM, *n* = 6. *p < 0.05 versus control group.

To evaluate the effect of oral administration of lead on body weight, repeated measures were used. The results showed that there was a statistically significant interaction between the effect of lead on body weight and weeks of lead administration [*F* (16, 100) = 59.07, *p* = 0.0001]. Tukey's post hoc test revealed a significant decrease (*p* < 0.05) in the body weight of rats of groups B, C, D, and E on week 3 and week 4 when compared to the body weight of rats in the control group. These results showed that the oral administration of lead for 4 weeks significantly reduced the weight of the rats (Figure 4).

### 3.5 Effect of Cur‐CSCaCO_3_NP on body weight of rats

**FIGURE 5:**
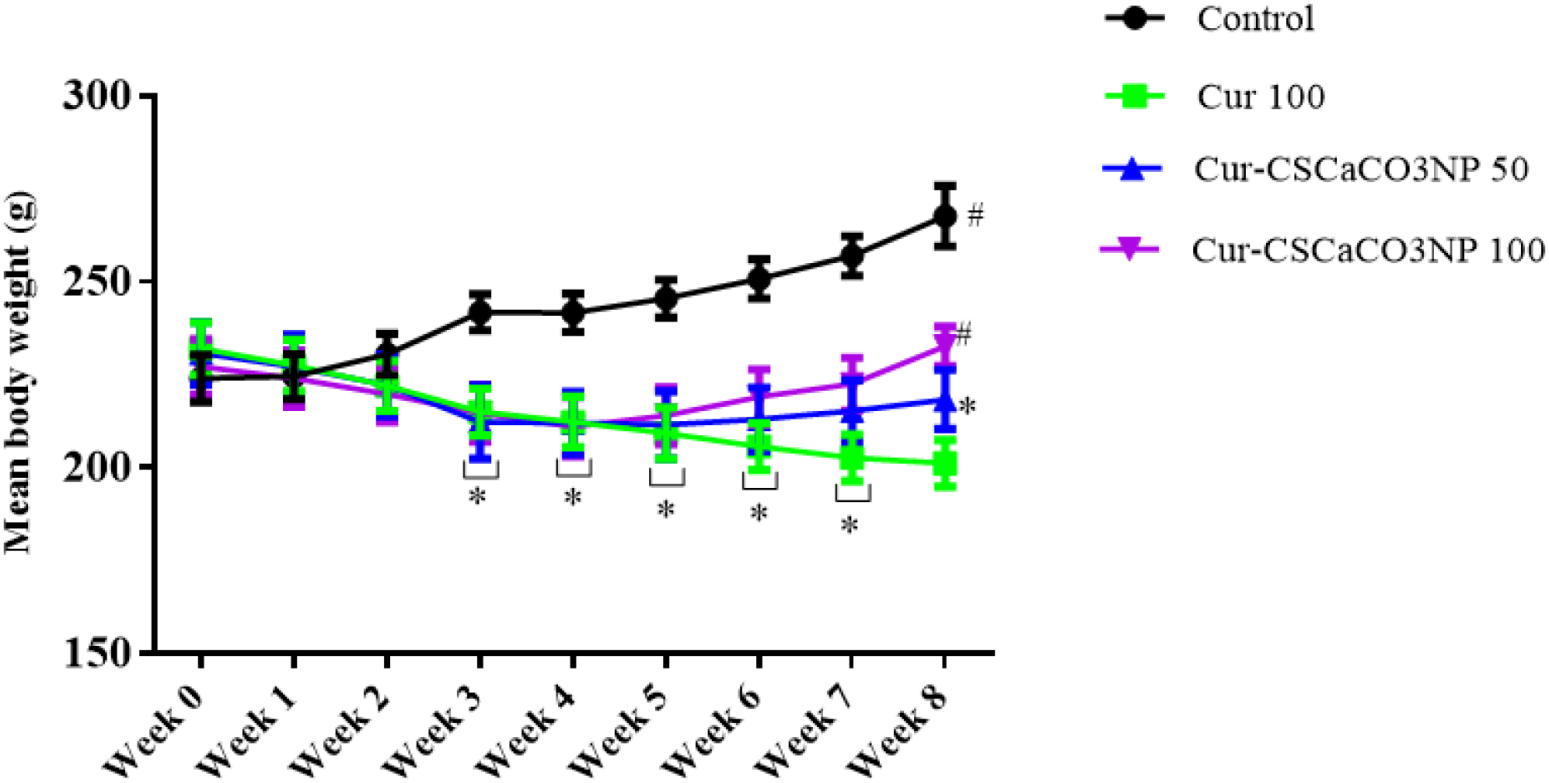
Effects of cur‐CSCaCO_3_NP and curcumin on the body weight of lead‐induced rats. Values were presented as mean ± SEM, *n* = 6. *p < 0.05 versus control group, #*p* < 0.05 versus Cur 100 group

To investigate the effect of Cur‐CSCaCO_3_NP and curcumin on lead‐induced rats, the trends of the body weight in all the various groups were analyzed. Repeated measures analysis revealed statistically significant interactions in the body weight of rats between the effect of treatment and weeks of treatment [*F* (24, 160) = 23.83, *p* = 0.0001]. Tukey's post hoc test revealed a significant decrease (*p* < 0.05) in the body weight of rats from Cur 100, Cur‐CSCaCO_3_NP 50, and Cur‐CSCaCO_3_NP 100 groups on week 3, week 4, week 5, week 6, and week 7 when compared to the body weight of rats in the control group. Furthermore, a similar trend was observed on week 8, where a statistically significant decrease (*p* < 0.05) in the body weight of rats in Cur 100 and Cur‐CSCaCO_3_NP 50 groups was observed when compared to the body weight of rats in the control group. Conversely, a statistically significant increase (*p* < 0.05) was observed in the body weight of rats in the control and Cur‐CSCaCO_3_NP 100 groups of rats when compared to the body weight of rats in the Cur 100 group (Figure 5).

### 3.6 Effects of lead on the weight of organs

**FIGURE 6:**
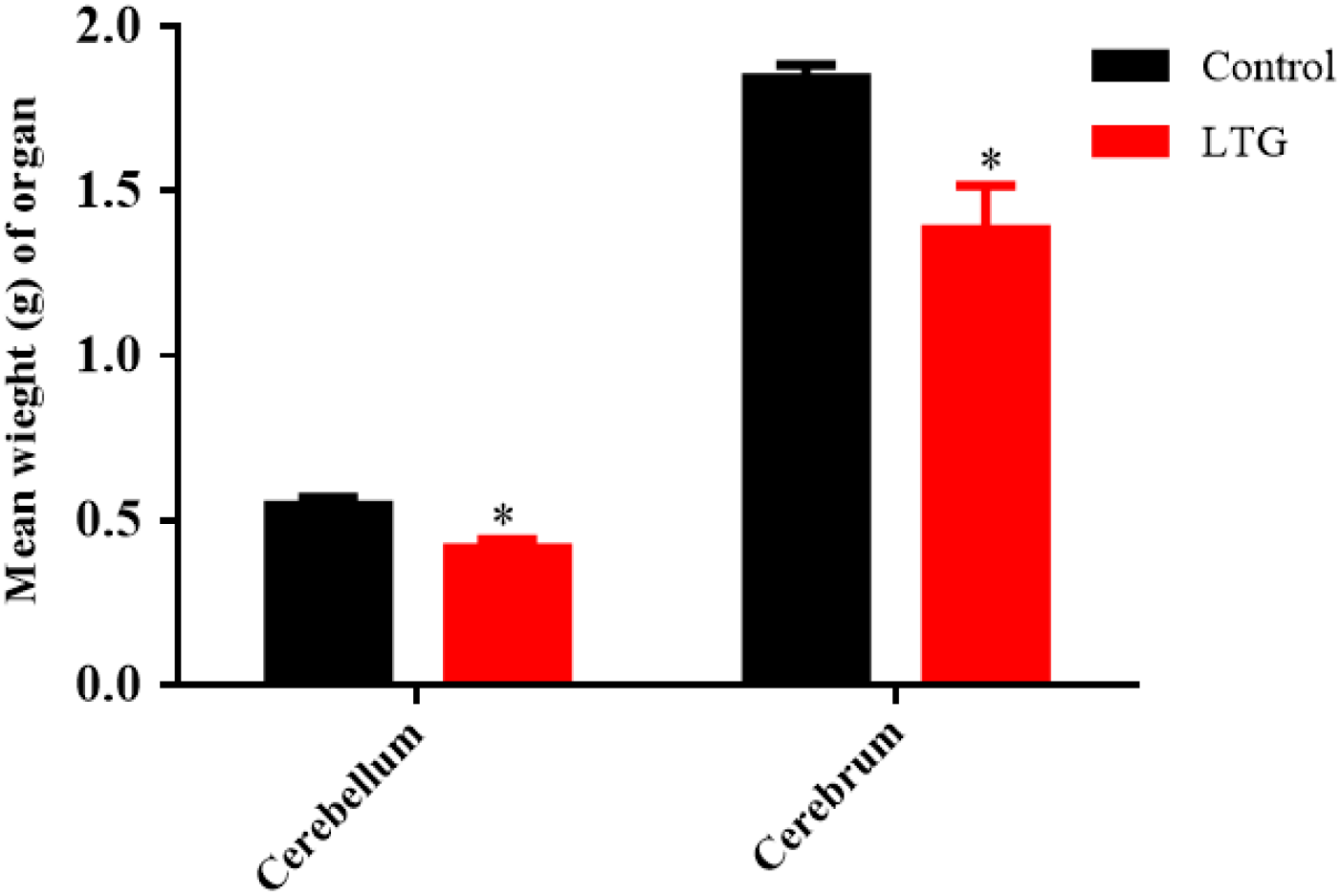
Effects of lead on the weight of organs in rats after 4 weeks of induction. Values were presented as mean ± SEM, *n* = 6. *p < 0.05 versus control group

Unpaired sample *t*‐tests were conducted to compare the weight of the cerebrum and cerebellum of rats treated with lead (50 mg/kg) and that of the control group. There was a significant difference between the weight of cerebellum of lead‐induced rats [LTG (0.4167 ± 0.02603)] and the cerebellum of the control group of rats (0.5450 ± 0.02432); conditions, *t* (10) = 3.602, *p* = 0.0048. Furthermore, a statistically significant difference between the weight of the cerebrum of lead‐induced rats [LTG (1.383 ± 0.1318)] and the cerebrum of the control group of rats (1.843 ± 0.03887), conditions, *t* (10) = 3.347, *p* = 0.0074, were observed. These results showed that lead significantly decreased the weights of the cerebellum and cerebrum of rats when compared to the control group (Figure 6).

### 3.7 Effects of Cur‐CSCaCO_3_NP on the weight of organs

**FIGURE 7:**
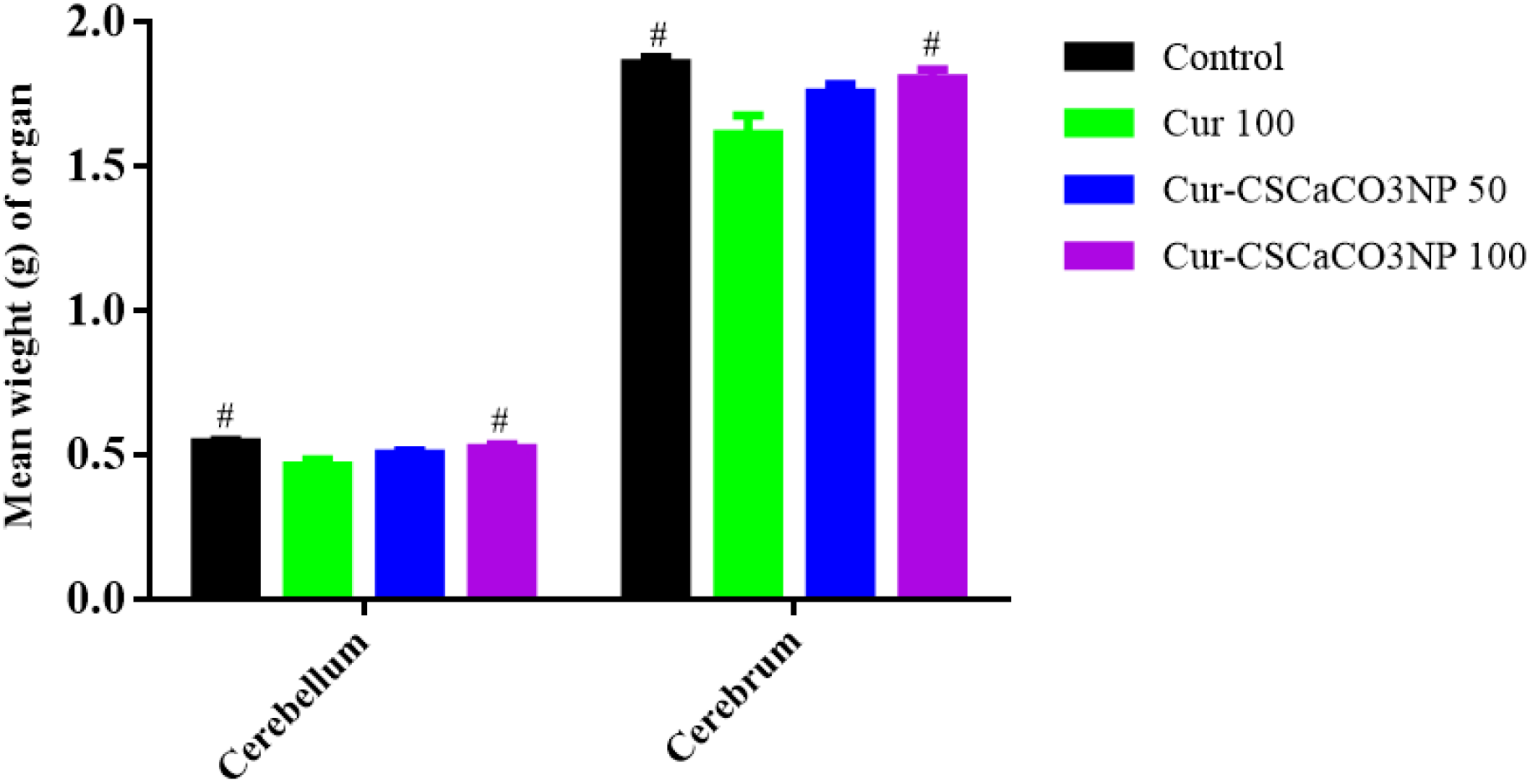
Effects of Cur‐CSCaCO_3_NP and curcumin on the weight of organs in rats exposed to lead. Values were presented as mean ± SEM, *n* = 6. #*p* < 0.05 versus Cur 100 group

As shown in Figure 7, one‐way ANOVA revealed statistically significant difference in the weights of the cerebellum and cerebrum [cerebellum: *F* (3, 20) = 5.805, *p* = 0.005, cerebrum: *F* (3, 20) = 7.019, *p* = 0.0021]. Tukey's post hoc revealed significant increases in the weight of the cerebellum of rats in the control groups (0.55 ± 0.010, *p* = 0.0039) and the group of rats treated with Cur‐CSCaCO_3_NP 100 (0.53 ± 0.012, *p* = 0.029), when compared to Cur 100 treatment group of rats (0.46 ± 0.022). Furthermore, a significant increase in the weight of the cerebrum of rats in the control group (1.86 ± 0.022, *p* = 0.0016) and the group of rats treated with Cur‐CSCaCO_3_NP 100 (1.8 ± 0.032, *p* = 0.013) when compared to Cur 100 treatment group (1.613 ± 0.064).

### 3.8 Effect of lead on SOD activities

**FIGURE 8:**
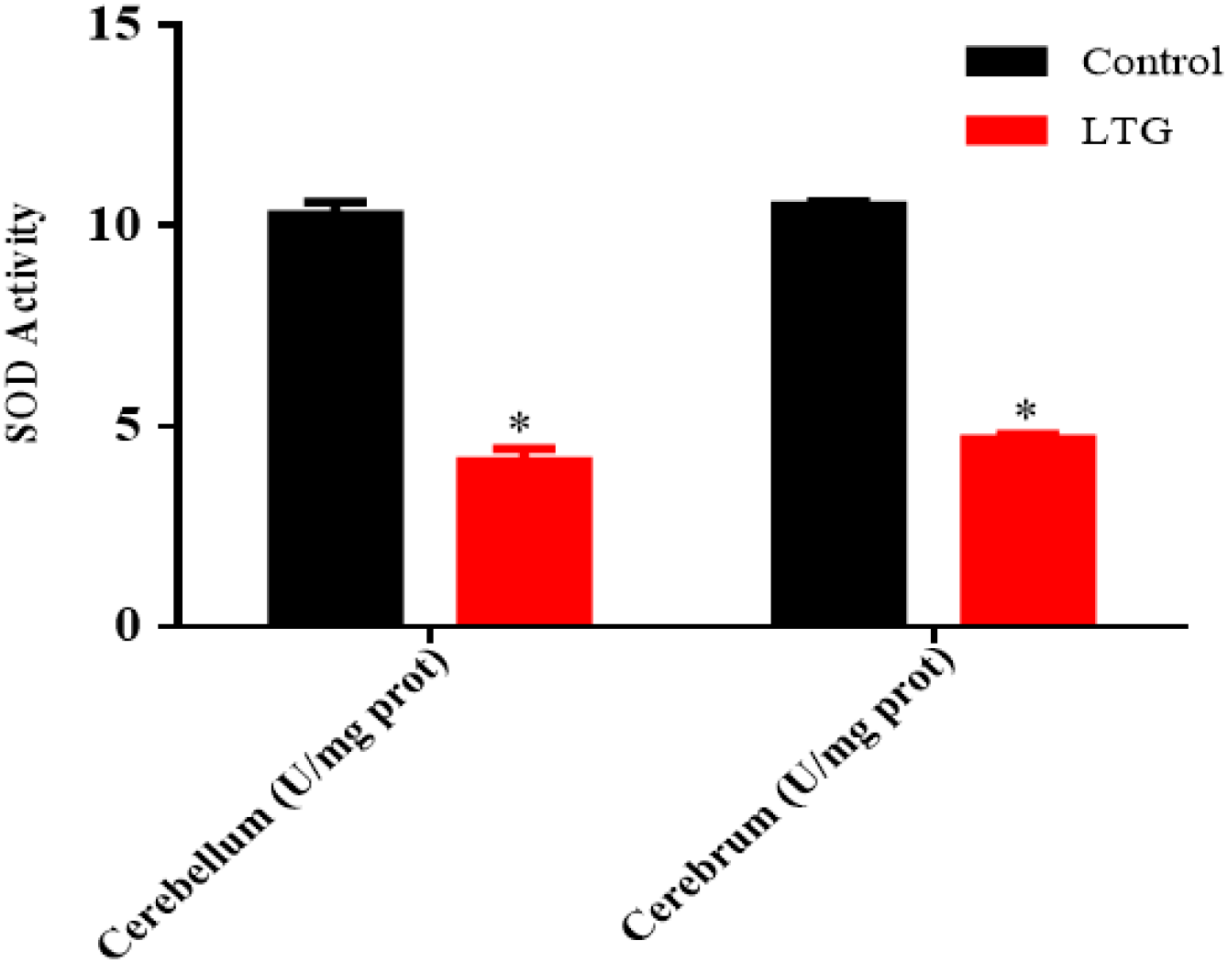
Effect of lead on superoxide dismutase (SOD) activities in the cerebellum and cerebrum of rats after 4 weeks of induction. Values were presented as mean ± SEM, *n* = 3. *p < 0.05 versus control

The activity of SOD in the cerebellum and cerebrum of the lead‐administered rats revealed statistically significant differences with the control group as shown by the unpaired independent *t*‐tests as follows: cerebrum [LTG (4.68 ± 0.13)] and the control groups of rats (10.50 ± 0.11); conditions, *t* (4) = 34.14, *p* = 0.0001. Furthermore, a statistically significant difference was observed in the cerebellum of lead‐induced [LTG (4.12 ± 0.32)] and the control groups of rats (10.28 ± 0.31); conditions, *t* (4) = 3.75, *p* = 0.0002. These results showed that lead significantly decreased the SOD activity in the cerebrum and cerebellum of the rats when compared to their respective controls (Figure 8).

### 3.9 Effect of Cur‐CSCaCO_3_NP on SOD activities

**FIGURE 9:**
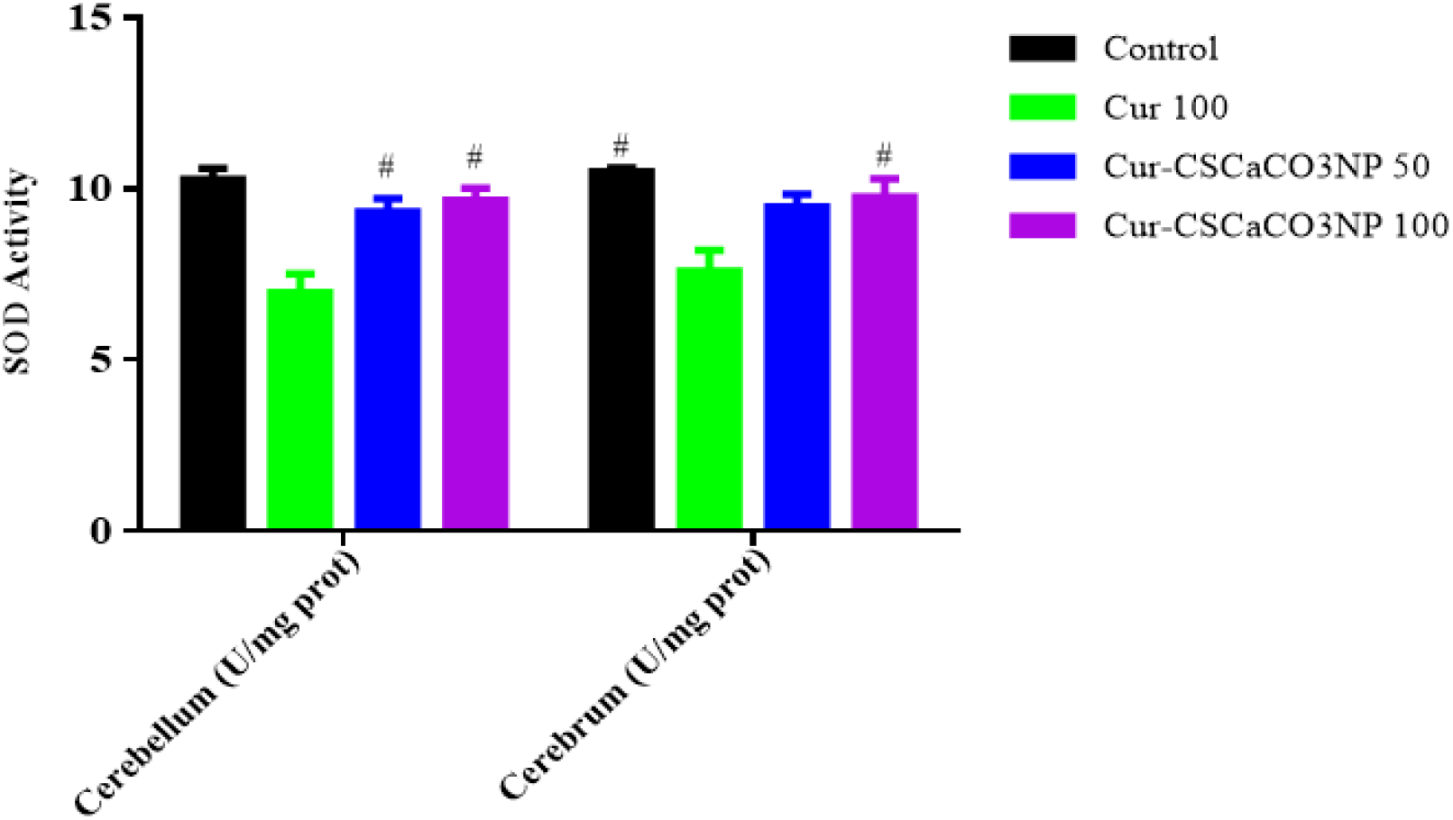
Effect of Cur‐CSCaCO_3_NP and curcumin on superoxide dismutase (SOD) activities in the cerebellum and cerebrum of lead‐administered rats after 4 weeks of treatment. Values were presented as mean ± SEM, *n* = 3. #*p* < 0.05 versus Cur 100.

As shown in Figure 9, the effect of Cur‐CSCaCO_3_NP and curcumin on the SOD activities of lead‐administered rats revealed statistically significant differences among the various rat groups as shown by the one‐way ANOVA as follows: cerebellum [*F* (3, 8) = 12.69 *p* = 0.0021] and cerebrum [*F* (3, 8) = 7.524, *p* = 0.0103]. The Tukey's post hoc showed statistically significant differences between the various groups as follows: a statistically significant increase in SOD was observed in the cerebellum control (10.28 ± 0.31, *p* = 0.0019), cerebellum Cur‐CSCaCO_3_NP 50 (9.33 ± 0.39, *p* = 0.0147), and cerebellum Cur‐CSCaCO_3_NP 100 groups of rats (9.67 ± 0.35, *p* = 0.0068) when compared to Cur 100 (6.99 ± 0.53). In addition, a statistically significant increase in SOD was observed in the cerebrum control group (10.50 ± 0.11, *p* = 0.0080), and the cerebrum Cur‐CSCaCO_3_NP 100 groups of rats (9.75 ± 0.54, *p* = 0.0384) when compared to Cur 100 (7.59 ± 0.61).

### 3.10 Effect of lead on MDA level

**FIGURE 10:**
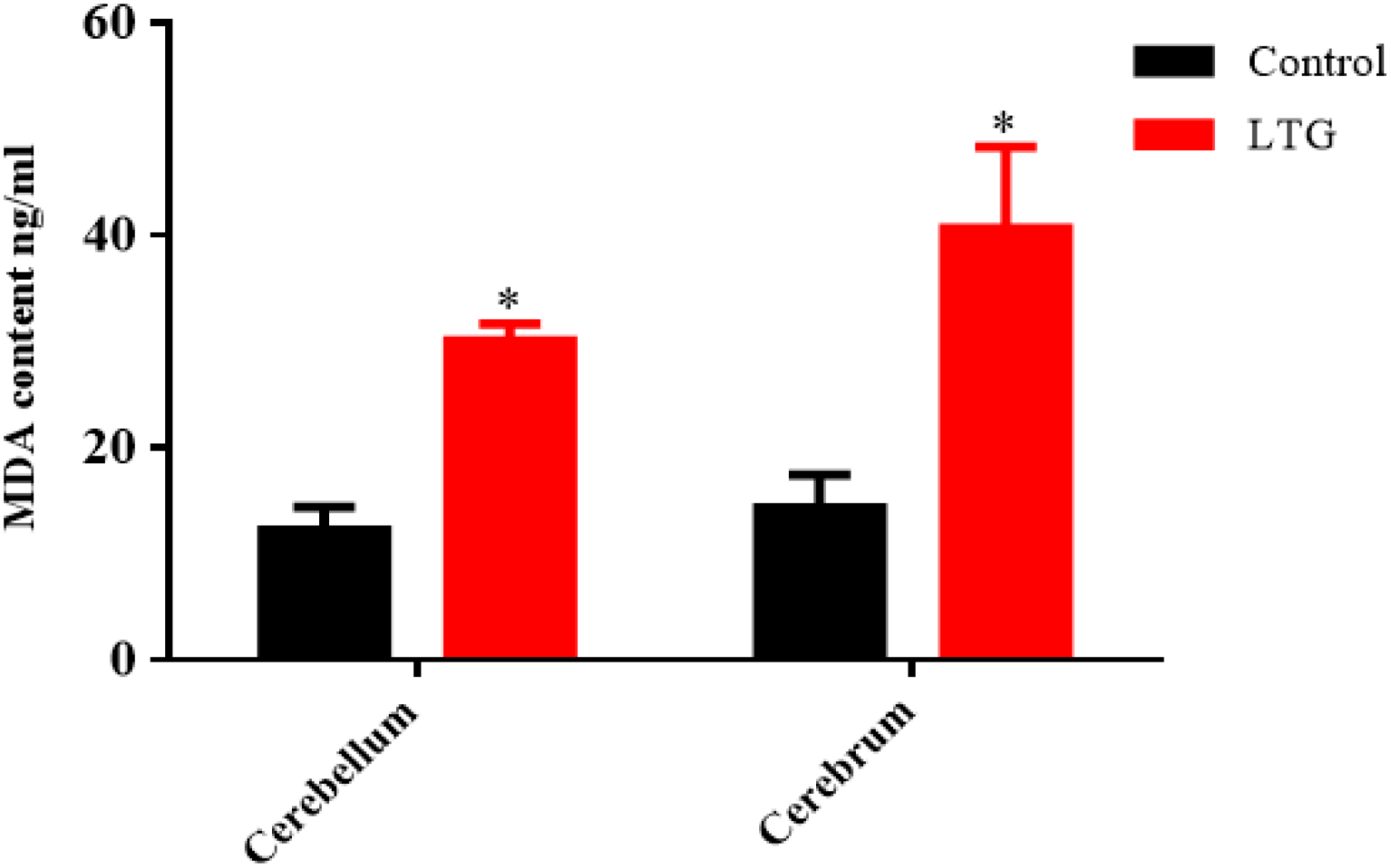
Effect of lead on malondialdehyde (MDA) levels in the cerebellum and cerebrum of rats after 4 weeks of induction. Values were presented as mean ± SEM, *n* = 3. *p < 0.05 versus control

The ELISA results of MDA level in the cerebellum and cerebrum of the lead‐administered rats revealed statistically significant differences with the control group as shown by the unpaired independent *t*‐tests as follows: cerebrum [LTG (40.69 ± 7.70)] and the control groups of rats (14.37 ± 3.09), conditions, *t* (4) = 3.174, *p* = 0.0337; and cerebellum [LTG (30.08 ± 1.57)] and the control groups of rats (12.29 ± 2.15), conditions, *t* (4) = 6.69, *p* = 0.0026. These results showed that lead significantly increased the MDA levels in the cerebrum and cerebellum of the lead‐induced rats when compared to their control groups (Figure 10).

### 3.11 Effect of Cur‐CSCaCO_3_NP on MDA level

**FIGURE 11:**
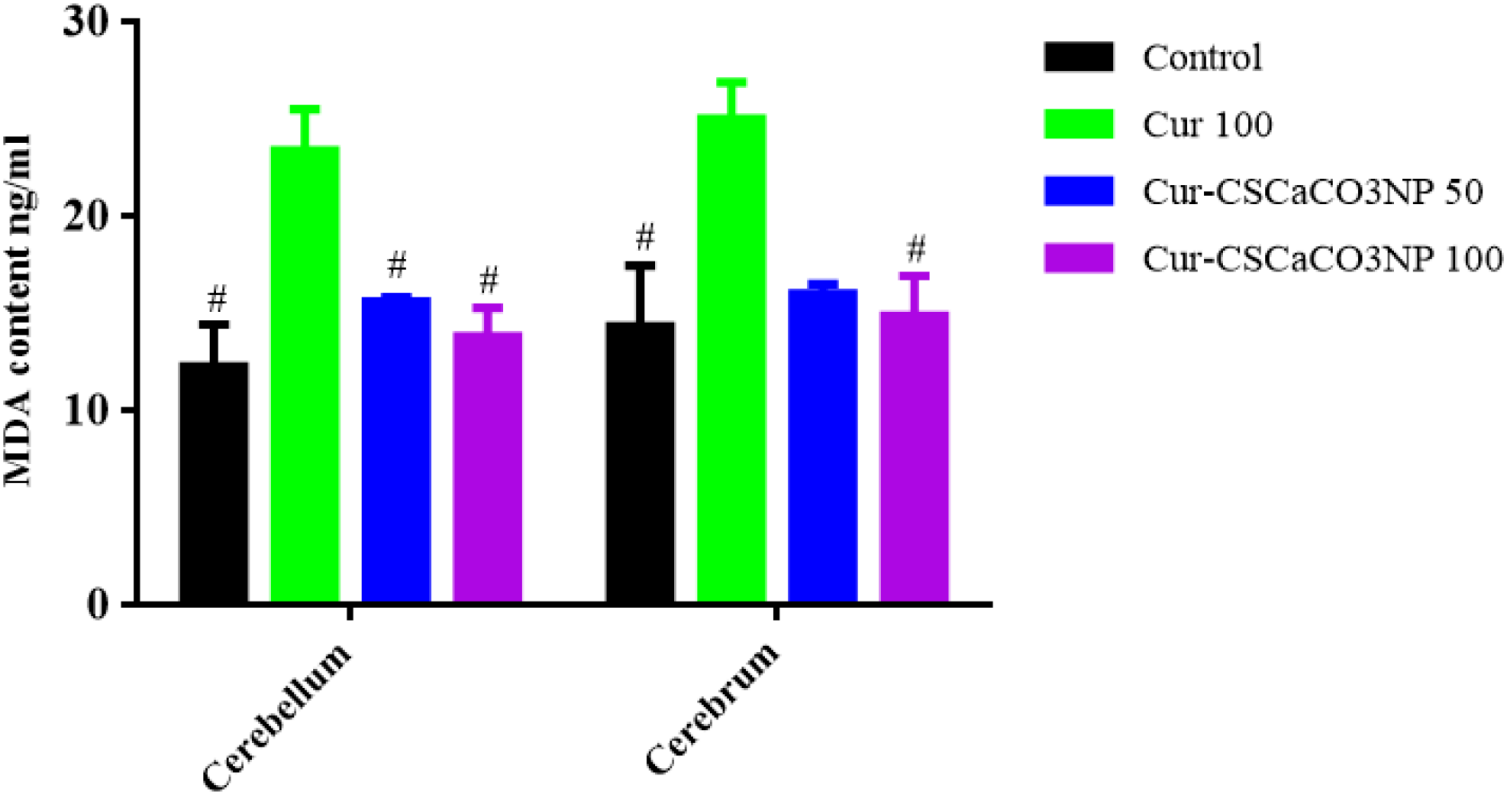
Effect of cur‐CSCaCO_3_NP and curcumin on malondialdehyde (MDA) levels in the cerebellum and cerebrum of lead‐administered rats after 4 weeks of treatment. Values were presented as mean ± SEM, *n* = 3, #*p* < 0.05 vs. cur 100.

As shown in Figure 11, based on the ELISA results, the one‐way ANOVA revealed statistically significant differences in MDA levels in the cerebellum and cerebrum among the rat groups as follows: [cerebellum: *F* (3, 8) = 8.625, *p* = 0.0069, cerebrum: *F* (3, 8) = 5.843, *p* = 0.0205]. The Tukey's post hoc showed statistically significant differences between the various groups as follows: a statistically significant decrease in MDA levels was observed in the cerebellum of the control group (12.29 ± 3.72, *p* = 0.007), the Cur‐CSCaCO_3_NP 50 group (15.61 ± 0.41, *p* = 0.05), and the Cur‐CSCaCO_3_NP 100 group (13.83 ± 2.53, *p* = 0.02) when compared to Cur 100 (23.40 ± 3.68). Furthermore, a statistically significant decrease in MDA levels was observed in the cerebrum of the control group (14.37 ± 5.35, *p* = 0.027) and Cur‐CSCaCO_3_NP 100 group (14.90 ± 5.54, *p* = 0.035) when compared to Cur 100 (25.05 ± 1.83).

## 4 HISTOPATHOLOGY

### 4.1 Histological examination of the cerebral cortex using H&E stain after lead induction

**FIGURE 12:**
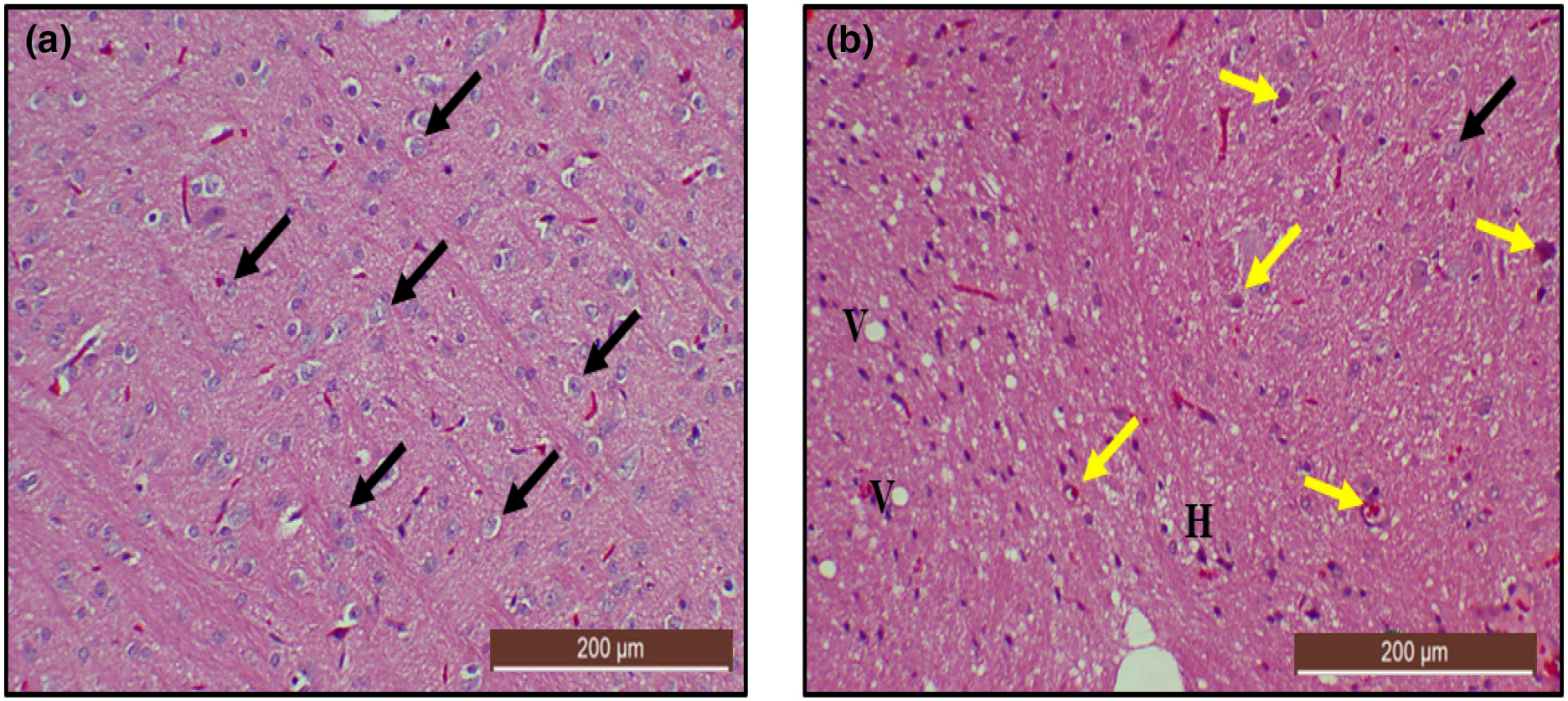
Hematoxylin and eosin (H&E)‐stained cerebral cortex sections from rats treated with (a) normal saline (control); showing the normal architectural and cellular morphology (black arrow) of the cerebral cortex (b) 50 mg/kg of lead (LTG) showing marked degenerations: hyperchromatic cells (yellow arrow), cellular atrophy, pyknosis, and vacuolated cells (V). (H&E, ×10 scale bar = 200 μm)

The histological section in Figure 12a showed the normal histological structure of the cerebral cortex and normal neuronal cell distributions and cellular morphology of the cerebral cortex in the control group of rats. However, the section in Figure 12b showed disorganized irregular neuronal cells indicating marked damage in the cerebral cortex compared to the control group of rats. Some prominent alterations were vacuolated cells, pyknosis, hyperchromatic cells, cellular atrophy, shrinkage, and cellular necrosis.

### 4.2 Histochemical examination of the cerebral cortex using toluidine blue stain after lead induction

**FIGURE 13:**
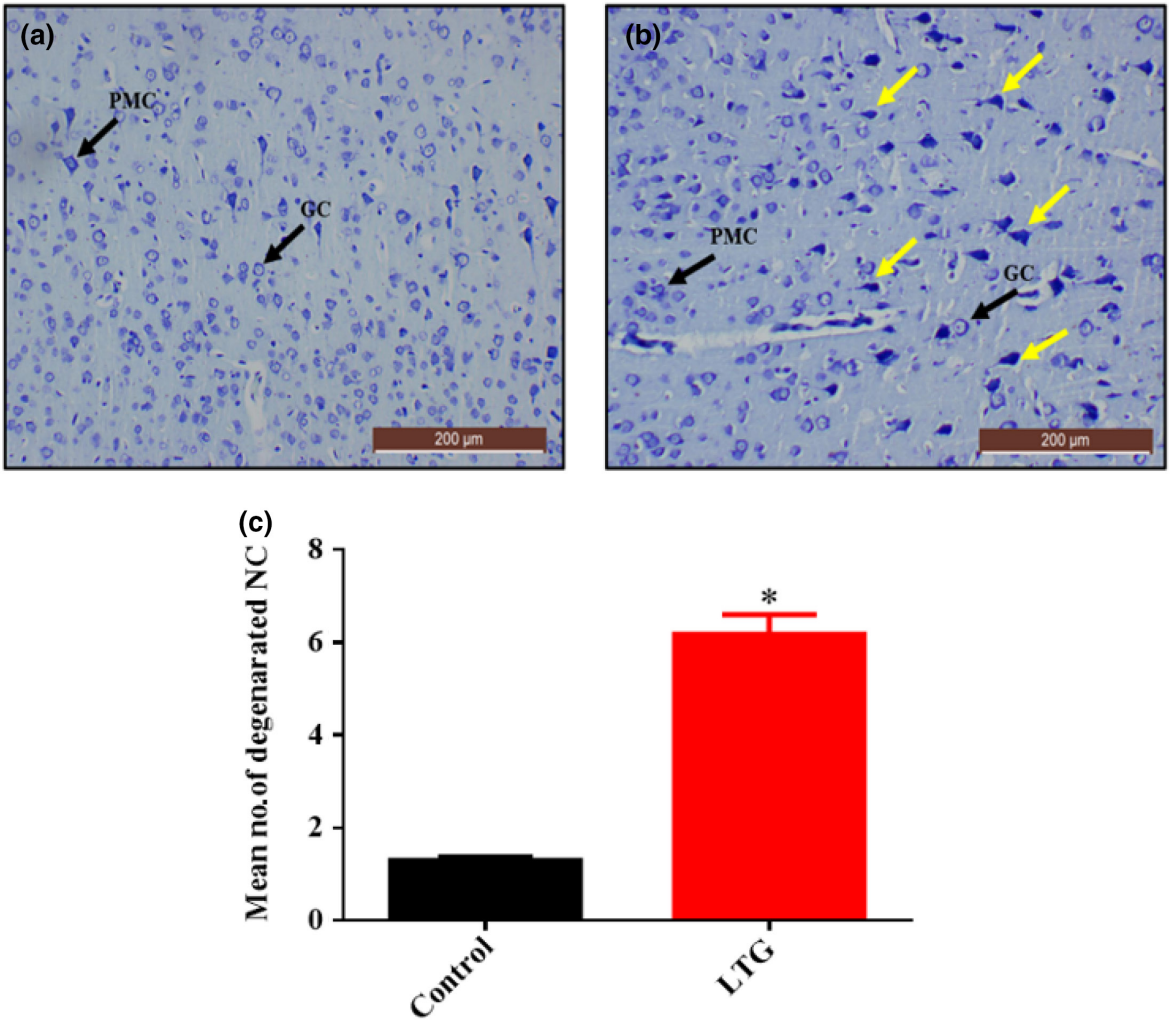
Toluidine blue‐stained cerebral cortex sections from rats treated with (a) normal saline (control); showing the array of well‐organized and normal morphology of the neuronal cells, granular cells (GC), and pyramidal cells (PMC) and (b) 50 mg/kg of lead (LTG) showing marked degenerations with prominent hyperchromatic cells (yellow arrow). (c) Quantitative representation of degenerated neuronal cells of the control and LTG groups. *p < 0.05 versus control, *n* = 5. (Toluidine blue, ×10, scale bar = 200 μm)

To further confirm the neurodegenerative effect of lead on the cerebrum of rats, a special stain was performed using a toluidine blue stain. The section from rats treated with lead (LTG) shows cellular degenerations with hyperchromatic neuronal cells (Figure 13b) when compared with a cerebral section of rats from the control group (Figure 13a). Furthermore, the nonparametric *t*‐test used for the quantification of nonviable neuronal cells revealed a statistically significant difference. Mann–Whitney test showed statistically significant increase in the number of non‐viable neuronal cells in LTG group (Mdn = 6.2), when compared to the control group (Mdn = 1.3), *U* = 0.0, *p* = 0.0022, as shown in Figure 13c.

### 4.3 Histological examination of the cerebral cortex using H&E stain after Cur‐CSCaCO_3_NP treatment

**FIGURE 14:**
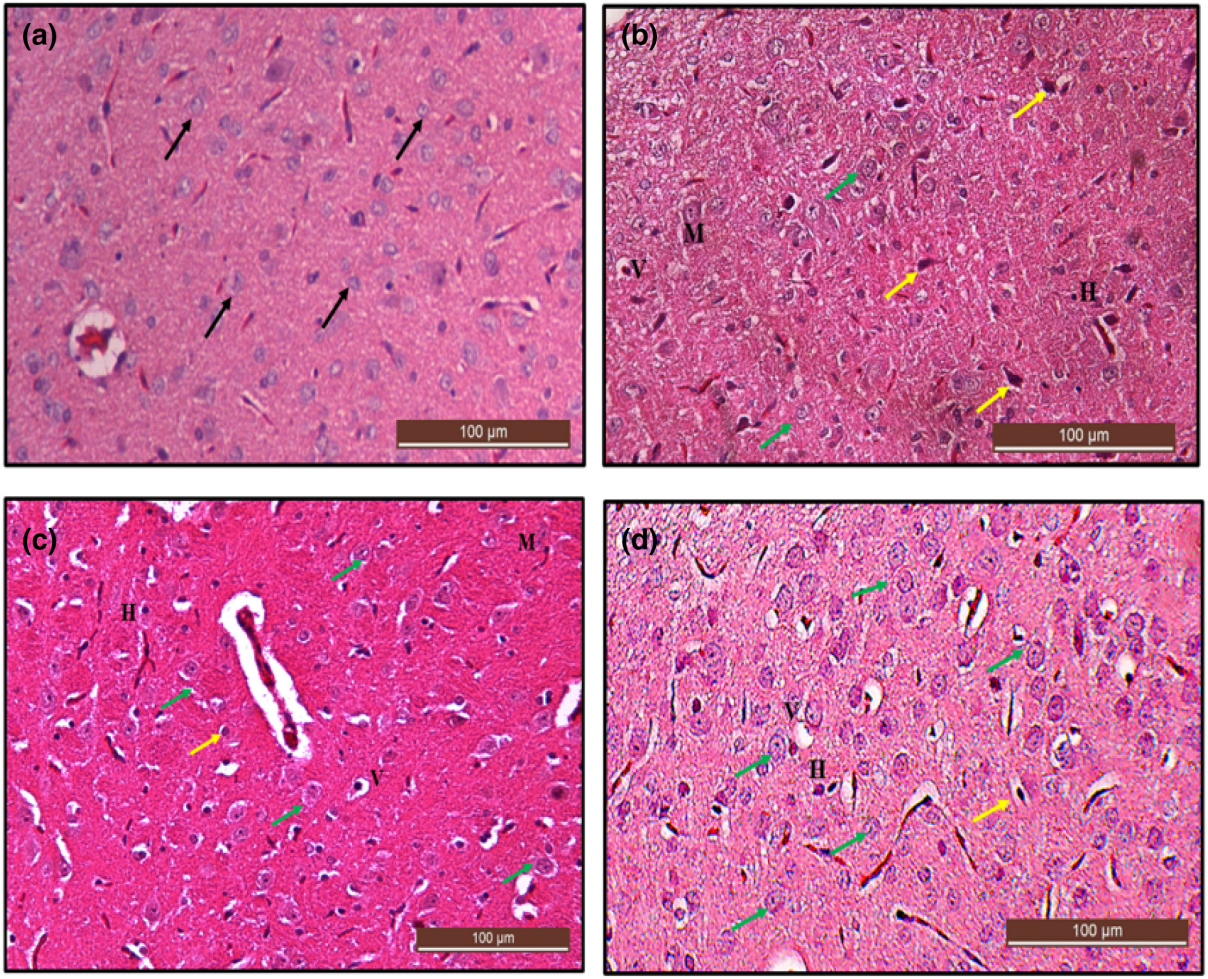
Hematoxylin and eosin (H&E)‐stained cerebral cortex sections from rats treated with (a) normal saline (control), showing the normal architectural and cellular morphology (black arrow) of the cerebral cortex. (b) 100 mg/kg of curcumin (cur 100) showing some level of cellular degenerations (yellow arrow), irregular‐shaped multipolar (M), and vacuolated neuronal cells (V) with hyperchromatic cells (H). (c) 50 mg/kg of cur‐CSCaCO_3_NP (cur‐CSCaCO_3_NP 50) and (d) 100 mg/kg of cur‐CSCaCO_3_NP (cur‐CSCaCO_3_NP 100) showing fewer damaged neuronal cells with marked improved normal neuronal cell morphology and very reduced vacuolar spaces around the cells (green arrow). (H&E, ×20, scale bar = 100 μm)

The histological section in Figure 14b showed that the neuronal cells had lost their characteristic shapes and appeared irregular. The neuronal cells appeared darkly stained with pyknotic nuclei. Some cells are multipolar with vacuolar space around them. Treatment with Cur‐CSCaCO_3_NP markedly decreased the neuropathological lesions as shown in Figure 14c,d when compared to section from Cur 100 group. Furthermore, the section from group Cur‐CSCaCO_3_NP (Figure 14d) showed marginal attenuation of the neuropathological changes caused by lead; thus, the histological features and cell distributions are like those in the control group (Figure 14a).

### 4.4 Histochemical examination of the cerebral cortex using toluidine blue stain after Cur‐CSCaCO_3_NP treatment

**FIGURE 15:**
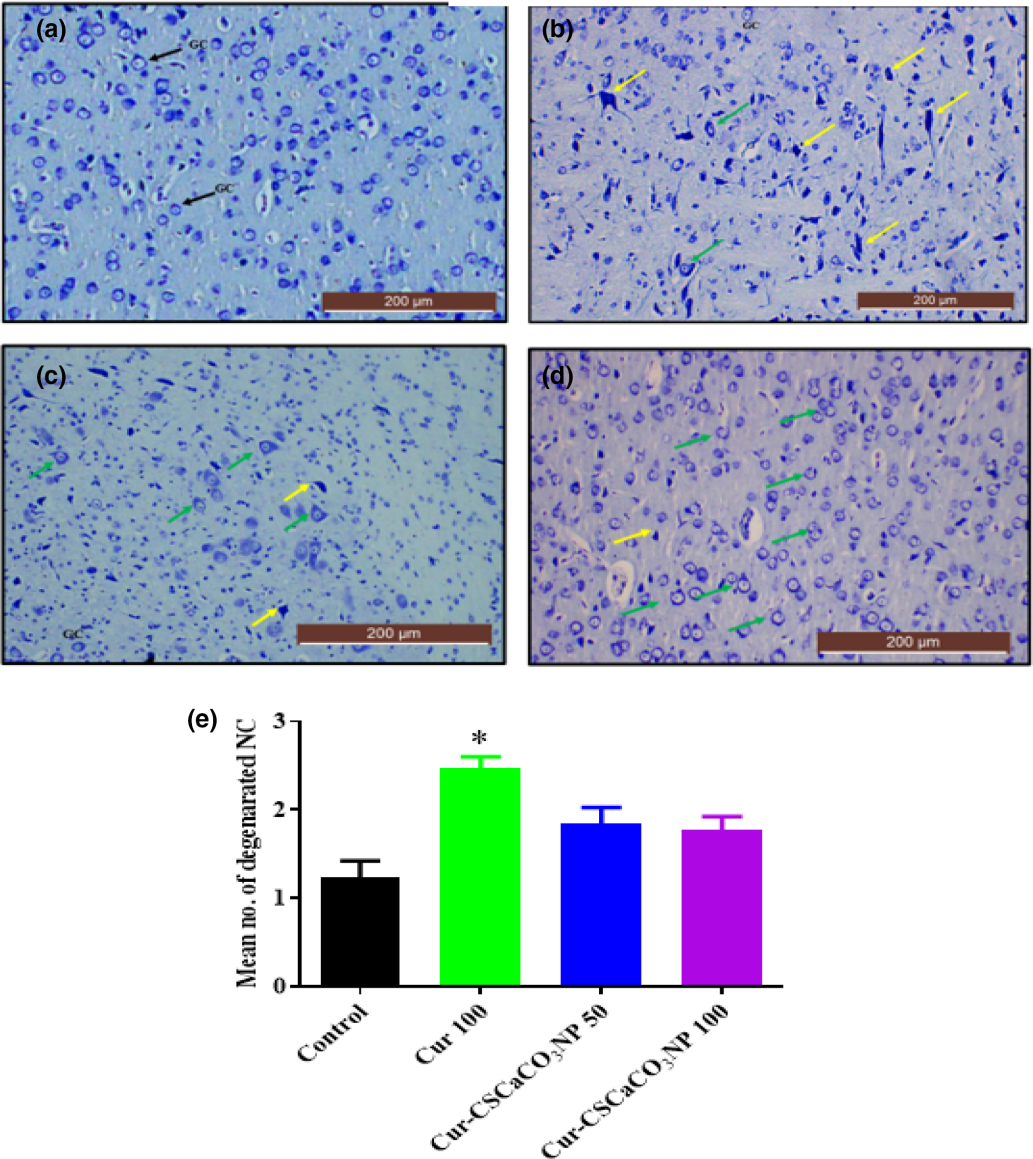
Toluidine blue‐stained cerebral cortex sections from rats treated with (a) normal saline (control); show the array of well‐organized and normal morphology of neuronal cells, granular cells (GC), and pyramidal cells (PMC). (b) 100 mg/kg of curcumin (cur 100) showing cellular degenerations: (c) cur‐CSCaCO_3_NP 50 and (d) cur‐CSCaCO_3_NP 100 showing fewer damaged neuronal cells with marked improved normal neuronal cells (green arrow). (e) Quantitative representation of degenerated neuronal cells of the control and all the treated groups. *p < 0.05 versus control, *n* = 5. (Toluidine blue, ×10, scale bar = 200 μm)

Further confirmation of the ameliorative effect of Cur‐CSCaCO_3_NP on lead‐induced cerebral damage was done using toluidine blue stain. The section from rats treated with free curcumin shows cellular degenerations with hyperchromatic neuronal cells and fewer healthy neuronal cells (Figure 15b) when compared with the cerebral section of rats from the control group (Figure 15a). In contrast, numerous viable neuronal cells were restored as seen in Figure 15c,d. Thus, Cur‐CSCaCO_3_NP attenuated the neuronal injury induced by lead. In addition, quantitative analysis of the non‐viable neuronal cells of the cerebral cortex revealed statistically significant difference [H (3) = 13.79, *p* = 0.0034]. The mean rank of control is 5.50, Cur 100 is 20.50, Cur‐CSCaCO_3_NP 50 is 12.67, and Cur‐CSCaCO_3_NP 100 is 11.33. Dunn's multiple‐comparison test further showed a statistically significant increase (2.47 ± 0.13, *p* = 0.0014) in degenerated neuronal cells in the cerebral cortex of rats from Cur 100 group of rats, when compared to the control group (Figure 15e).

### 4.5 Histological examination of the cerebellum using H&E stain after lead induction

**FIGURE 16:**
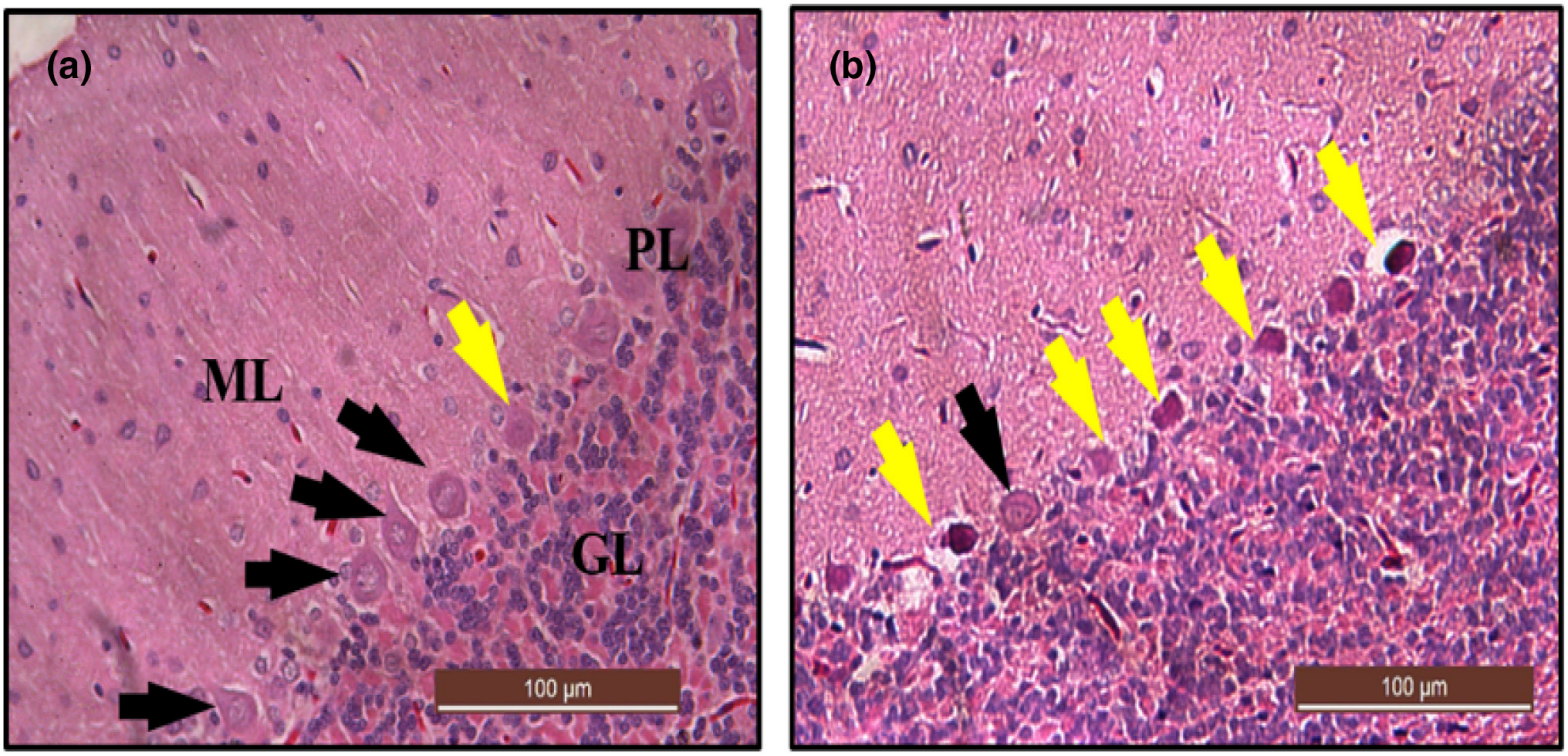
Photomicrograph of cerebellar sections from rats treated with (a) normal saline (control) showing normal histoarchitecture of the cerebrum with abundant healthy viable Purkinje cells (black arrow) and normal molecular layer (ML), Purkinje cells layer (PL), and granular layer (GL). (b) 50 mg/kg of lead (LTG) showing marked degeneration of Purkinje cells (yellow arrow). (H&E, ×20, scale bar = 100 μm).

Sections in Figure 16a showed a normal morphological appearance of the cerebellum under a light microscope. The outer gray matter revealed three distinct layers, the outer molecular layer with glia cells, middle Purkinje cell layer with pyriform‐shaped Purkinje cells, and inner granular layer with deeply stained granule cells. In contrast, a section of the cerebellum from the LTG group of rats showed marked alterations in the area of degenerated Purkinje cells. Further prominent alterations including cellular shrinkage, scattered glia cells, and hyperchromatic Purkinje cells appeared to be surrounded by vacuolar space (Figure 16b).

### 4.6 Histochemical examination of the cerebellum using toluidine blue stain after lead induction

**FIGURE 17:**
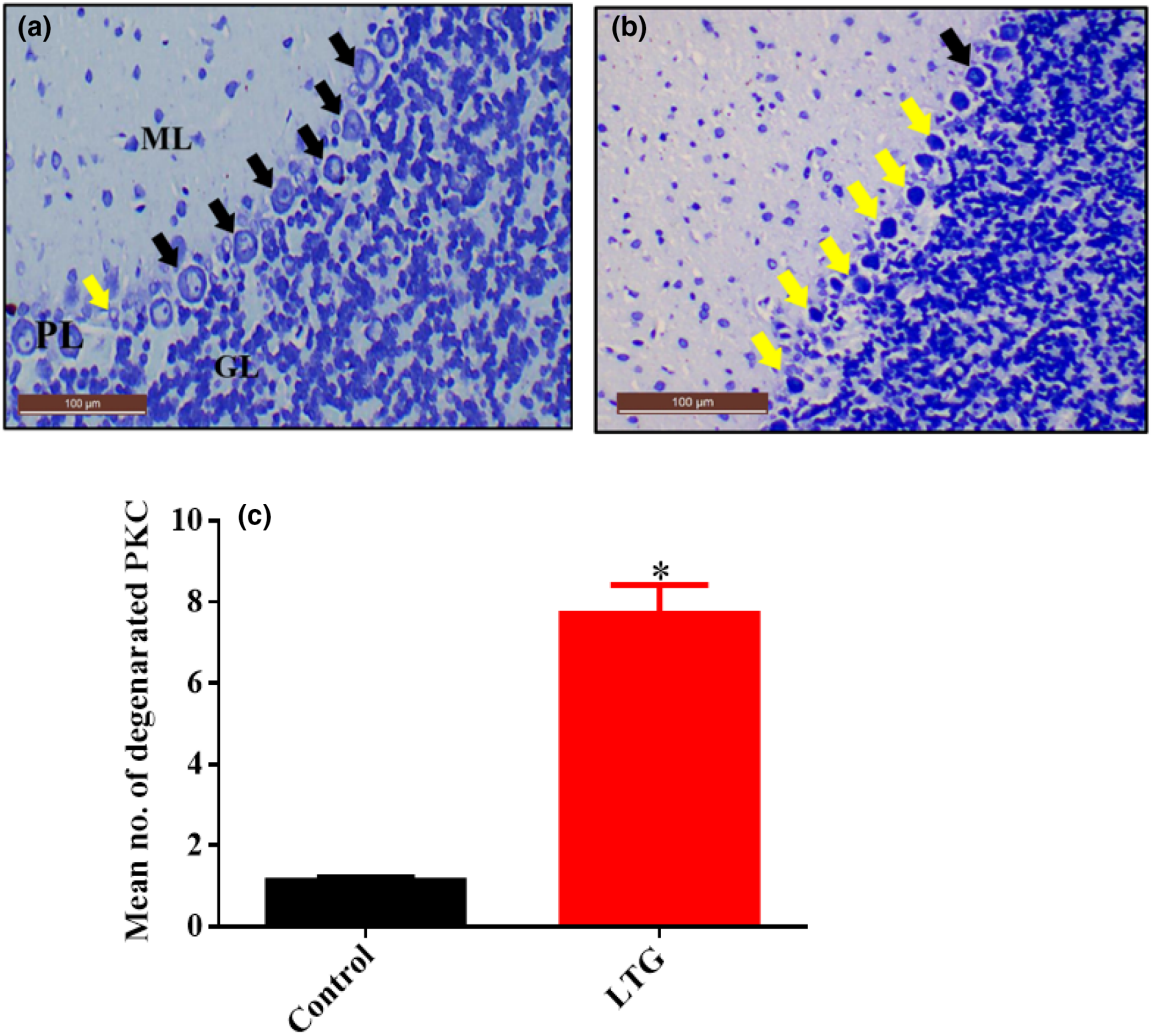
Photomicrograph of cerebellar sections from rats treated with (a) normal saline (control) showing normal histoarchitecture of the cerebellum with viable Purkinje cells (black arrow) and normal molecular layer (ML), Purkinje cells layer (PL), and granular layer (GL). (b) 50 mg/kg of lead (LTG) showing marked degeneration of Purkinje cells (yellow arrow). (c) Quantitative representation of degenerated neuronal cells of the control and LTG groups. *p < 0.05 versus control, *n* = 5. (Toluidine blue, ×20, scale bar = 100 μm)

The toluidine blue‐stained section from rats treated with lead (LTG) showed cellular degenerations with distortion of the Purkinje cells layer. The Purkinje cells appeared to be irregular in shape with darkly stained cytoplasm and distorted nuclei (Figure 17b) when compared with the normal cerebellar morphology of the control group (Figure 17a). Furthermore, the non‐parametric t‐test used for the quantification of number of degenerated Purkinje cells revealed a statistically significant difference. Mann–Whitney test showed a statistically significant increase in the number of degenerated Purkinje cells in the LTG group (Mdn = 7.7) when compared to the control group (Mdn = 1.15), *U* = 0.0, *p* = 0.0022, as shown in Figure 17c.

### 4.7 Histological examination of the cerebellum using H&E stain after Cur‐CSCaCO_3_NP treatment

**FIGURE 18:**
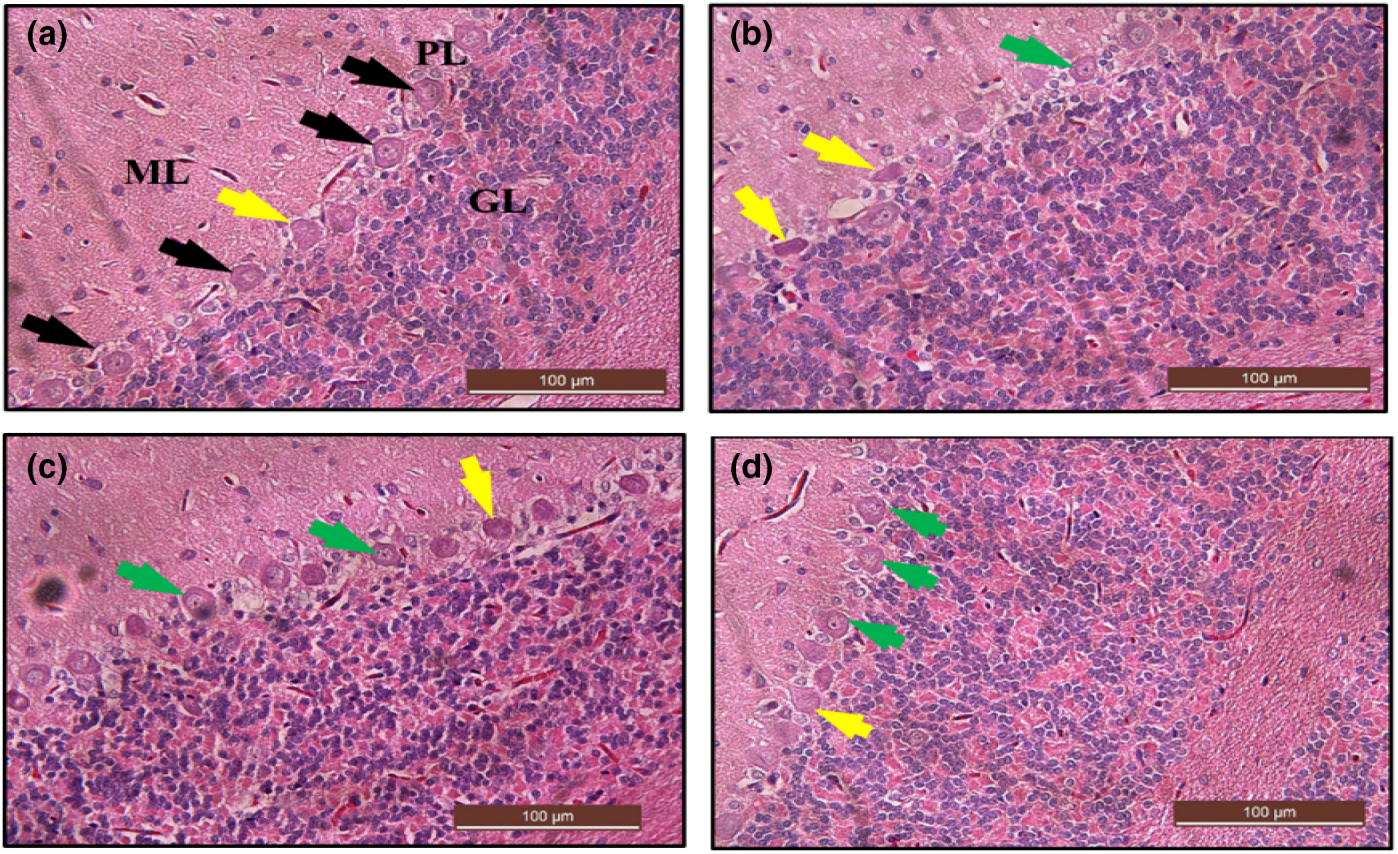
Hematoxylin and eosin (H&E)‐stained cerebellum sections from rats treated with (a) normal saline (control) showing the normal layers architecture molecular layer (ML), Purkinje layer (PL), and granular layer (GL): normal piriform‐shaped Purkinje cells (black arrow). (b) 100 mg/kg of curcumin (Cur 100) showing vacuolar spaces and irregular Purkinje cells with eosinophilic cytoplasm (yellow arrow). (c) 50 mg/kg of Cur‐CSCaCO_3_NP (Cur‐CSCaCO_3_NP 50) shows fewer damaged Purkinje cells and very reduced vacuolar spaces around the cells. (d) 100 mg/kg of Cur‐CSCaCO_3_NP (Cur‐CSCaCO_3_NP 100) showing marked improved normal Purkinje cell morphology with prominent nuclei (green arrow). (H&E, ×20, scale bar = 100 μm)

The cerebellar sections of rats in Cur 100 revealed scattered glial cells with vacuolar space in the molecular layer. Although the granular layer appeared to have a normal histological appearance (Figure 18b) when compared to the control group (Figure 18a), sections of the Cur‐CSCaCO_3_NP 50 and Cur‐CSCaCO_3_NP 100 cerebellar cortex showed restoration of the layers of the cerebellum after morphological alterations caused by lead. The Purkinje cells appeared to be regular in form with prominent nuclei and restored healthy cerebellar layers with regular pyriform‐shaped Purkinje cells as shown in Figure 18c,d.

### 4.8 Histochemical examination of the cerebellum using toluidine blue stain after Cur‐CSCaCO_3_NP treatment

**FIGURE 19:**
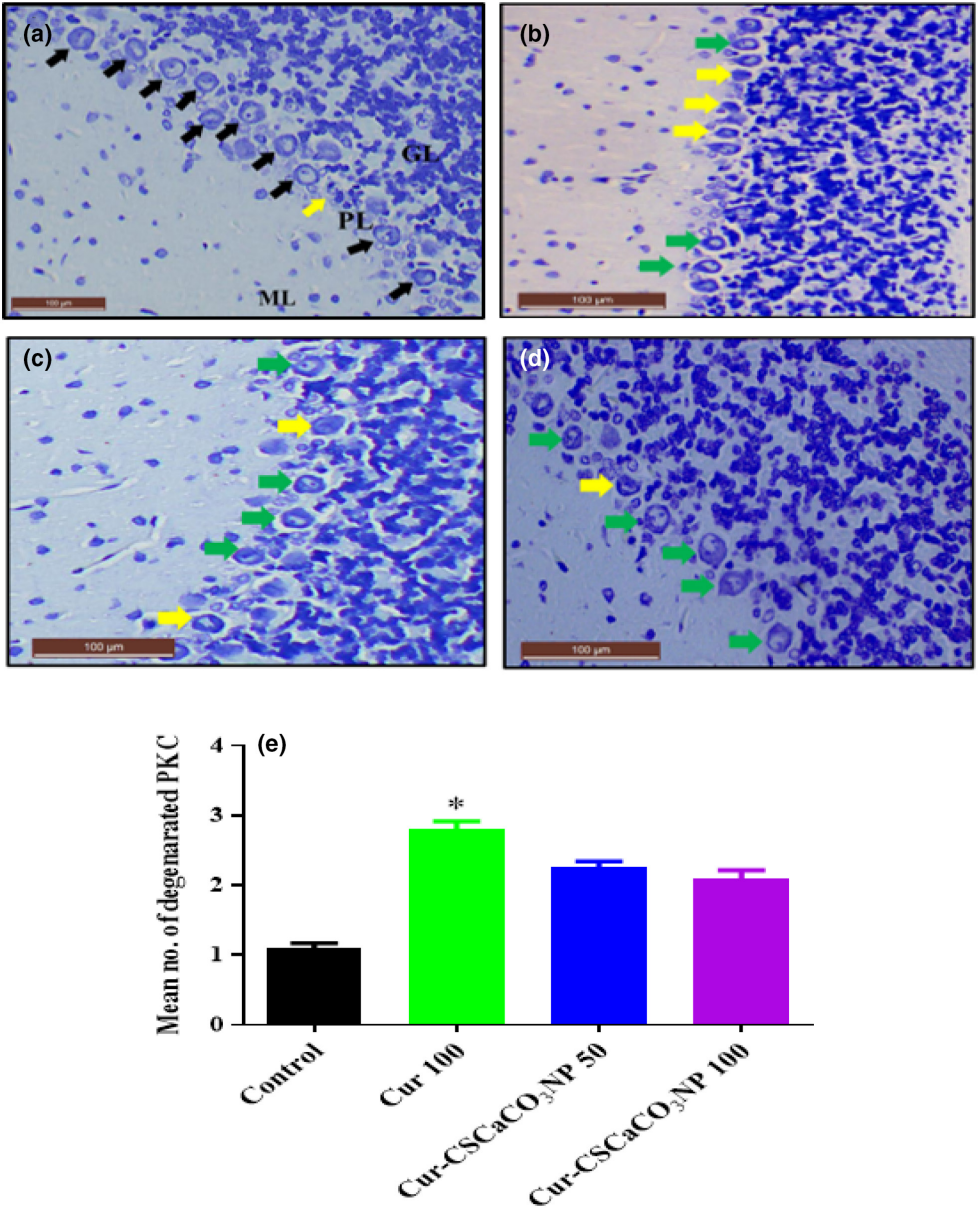
Toluidine blue‐stained cerebellum sections from rats treated with (a) normal saline (control) showing the normal layers architecture molecular layer (ML), Purkinje layer (PL), and granular layer (GL): And normal piriform‐shaped Purkinje cells (black arrow). (b) 100 mg/kg of curcumin (cur 100) showing vacuolar spaces and irregular Purkinje cells with eosinophilic cytoplasm (yellow arrow). (c) 50 mg/kg of cur‐CSCaCO_3_NP (cur‐CSCaCO_3_NP 50) showing fewer damaged Purkinje cells. (d) 100 mg/kg of cur‐CSCaCO_3_NP (cur‐CSCaCO_3_NP 100) showing marked improved layers organization and restoration of normal Purkinje cell morphology (green arrow). (e) Quantitative representation of degenerated Purkinje cells of the control and all the treated groups. *p < 0.05 versus control, *n* = 5. (Toluidine blue, ×20, scale bar = 100).

Histochemical examination of the sections of the cerebellum from rats of Cur 100 showed alteration in the Purkinje cell layer with few degenerated Purkinje cells. However, the molecular and granular layers appeared to be normal (Figure 19b). Furthermore, the cerebellar section from the Cur‐CSCaCO_3_NP 50 and Cur‐CSCaCO_3_NP 100 showed restoration of the healthy Purkinje cells. The molecular, Purkinje, and granular layers appeared to be normal as shown in Figure 19c,d when compared to the normal organized morphological features of the section from the control group of rats (Figure 19a). In addition, semiquantitative analysis of the nonviable neuronal cells of the cerebral cortex revealed statistically significant difference [H (3) = 18.38, *p* = 0.0004]. The mean rank of control is 3.5, Cur 100 is 20.75, Cur‐CSCaCO_3_NP 50 is 14.08, and Cur‐CSCaCO_3_NP 100 is 11.67. Dunn's multiple‐comparison test further showed a statistically significant increase (2.8 ± 0.12, *p* = 0.0001) in degenerated Purkinje cells in the cerebellum of rats from the Cur 100 group of rats, when compared to the control group (Figure 19e).

## 5 DISCUSSION

Previous studies on rats and humans have shown that exposure to lead could induce a series of pathological alterations resulting in serious health implications, manifesting different pernicious effects on multiple organs, particularly the brain (Abubakar, Mailafiya, et al., 2019; Rehman et al., 2018). These pernicious effects caused by lead intoxications may include redox homeostasis imbalance (Seddik et al., 2010; Sidhu & Nehru, 2004), cognitive impairments (Carrington et al., 2019), loss of motor coordination and paralysis (Mason et al., 2014), dullness and loss of memory (Vlasak et al., 2019), degeneration of neuronal cells, alteration of astrocyte maturation (Husain, 2015), and loss of integrity of blood–brain barrier (Beata et al., 2007). In this study, lead induction in rats revealed marked loss of brain and body weights. In addition, poor performance in horizontal bar test for motor functions, oxidative stress, marked degeneration of pyramidal cells of the cerebrum, and Purkinje cells in the cerebellum with generalized histopathological degenerations observed in the cerebrum and cerebral tissues. Although treatment with free curcumin to some extent reversed the aforementioned toxic damages induced by lead in the rats, significant ameliorative effects were observed by Cur‐CSCaCO_3_NP (50 and 100 mg/kg) treatments. However, the treatment was more effective at a higher dose of 100 mg/kg (Cur‐CSCaCO_3_NP 100).

Excellent cognitive function and coordination of motor behavior are linked to the core psychological functions and the integrity of the nervous system (Cecil et al., 2008). The neuronal cells, especially the Purkinje cells, are primarily attacked by environmental toxins such as lead upon exposure which consequently resulted in injury and eventual cell degeneration (Husain, 2015). The choice for a horizontal bar method is because this study stressed the effect of Cur‐CSCaCO_3_NP on lead‐induced toxicity on the cerebellum (responsible for balancing and coordination) and cerebrum (responsible for learning and voluntary movements). Thus, the horizontal bar test is designed for the assessment of strength and coordination that requires the process of learning for successful performance (Adolph & Franchak, 2017; Deacon, 2013; Ivens & Machemerf, 1998). On the account of this, the motor functions of rats in the control group improved by the subsequent weeks in this study, owing to their excellent learning skills, which contributed to their strong motor coordination. However, a progressively significant decrease in motor function and poor learning process was observed in rats administered with lead. This is in agreement with the previous work of Nehru and Sidhu (2002), who reported poor learning performance in measurement of motor coordination skills in lead‐exposed rats, and thus, concluded that lead exposure produces behavioral and motor coordination disturbances which are associated with dopaminergic and cholinergic neurotransmission in the CNS. In addition, previous studies also reported a decrease in cognitive and motor functions in rats exposed to lead (Azzaoui et al., 2009; Luthman et al., 1992; Mason et al., 2014; Sabbar et al., 2018). Conversely, the present study observed improvements in motor function of the rats treated with free curcumin and a better improvement in the motor function of the rats treated with Cur‐CaCO_3_NP. This could be attributed to the potential ability of the nanocarrier to enhance the efficacy of free curcumin against lead‐induced toxicity, thereby improving its therapeutic actions. Similar findings were reported in the work of Kakkar and Kaur (2011), who reported the ability of curcumin‐loaded solid lipid nanoparticles in alleviating behavioral changes induced by aluminum chloride. In addition, Sun et al. (2017) reported 15% recovery for free curcumin and 97.46% recovery for curcumin‐loaded solid lipid nanoparticles in attenuating cognitive deficit and behavioral changes in mice models of Alzheimer's disease. Furthermore, Moore et al. (2019) reported that chronic oral administration of curcumin alleviates neuroinflammations, thereby improving motor functions in middle‐aged rhesus monkeys. Chongtham and Agrawal (2016) also documented the ability of curcumin to alleviate Huntington's disease by modulating cell death owing to its anti‐inflammatory and antioxidant properties.

There are reports on the loss of body weights with resultant organ damage in animals continuously exposed to heavy metals such as lead (Alwaleedi, 2016; Amjad et al., 2013; Kabeer et al., 2019). In the present study, the observed decrease in the brain and body weights of lead‐administered rats were due to the toxic effect manifestation of lead exposure in the rats. The significant reduction in body weight markedly increased with the duration of oral lead administration, which explained the progressive body weight reduction observed in later weeks (week 4) of this study. Thus, the weight loss observed might be associated with the lead ability to interrupt the absorption and metabolism feed nutrients, which is impactful to health as reported earlier by Alwaleedi (2016). Body weight reduction in lead‐induced toxicity was reported in previous literatures (Abdel Moneim et al., 2011; Khan et al., 2008; Varnai et al., 2004). However, treatment with curcumin and Cur‐CSCaCO_3_NP showed body weight gain in the animals and stabilized their diet condition, although body weight gain was more obvious in rats treated with Cur‐CSCaCO_3_NP. This is because Cur‐CSCaCO_3_NP demonstrated an enhanced efficacy on the lead‐induced rats, and thus, stabilizing their body weights and improved the rats' condition as well as their diet condition. This is in accordance with previous literatures which documented improved diet conditions and an increase in body weight after treatment with both free curcumin and curcumin‐loaded nanoparticles (Abdel et al., 2022; Husain, 2015). Another possible reason could be due to CSCaCO_3_NP consisting of calcium, which may actually contribute to the increase in bone density that may consequently constitute to increase in rats' weight since body weight is directly associated with bone mineral density as reported by previous literatures. (Hoque et al., 2014; Jaji, Abu Bakar, et al., 2017; Liu et al., 2019; Mohd Abd Ghafar et al., 2017; Sukumar & Shapses, 2012)

One of the major consequences of lead‐induced toxicity is oxidative stress which reflects an imbalance of oxidative status due to continuous free radicals liberation and insufficient antioxidant activity to detoxify the resulting damage (Offor et al., 2017). Thus, lead can cause oxidative damage via two mechanisms operating simultaneously, which are overproduction of ROS and depletion of antioxidant reserves (Flora et al., 2012). These liberated ROS causes damage to cells by acting directly on lipid membranes resulting in lipid peroxidation (Wani et al., 2015). In this study, lead administration significantly decreases SOD activities and increases the MDA levels in the rats' serum, as well as cerebral and cerebellar tissue homogenates, respectively. This could be attributed to the ability of lead to induce the continuous generation of reactive oxygen species and general alterations of the system ROS scavenging enzymes (antioxidant) leading to cell membrane damage. This is in accordance with the work of Hamza et al. (2017), who reported that lead exhibits significant inhibitions of antioxidant enzymes' activities, thus, inducing oxidative stress in rats leading to free radicals' generation. Offor et al. (2017) observed a significant reduction in SOD activities with an increase in MDA level in rats under the influence of lead. In addition, other previous literatures reported a decrease in SOD activity and an increase in MDA level in brain tissues of rats due to lead exposure (Lakshmi et al., 2013; Sidhu & Nehru, 2004; Velaga et al., 2014). However, in this study, treatment with free curcumin to some extent demonstrated some healing process which is in agreement with the work of Abubakar, Muhammad Mailafiya, et al., (2019), who reported that curcumin attenuates lead‐induced neurotoxicity in rats via inhibition of oxidative stress and chelating activity. However, in this study, treatment with Cur‐CSCaCO_3_NP demonstrated excellent oxidative stress inhibition by significantly reversing the altered activities of SOD and conversely lowered the MDA level when compared to the free curcumin treatments. This could be due to the enhanced effect of the nanocarrier on curcumin; thus, improving its antioxidant effect. Other literatures documented the combined antioxidant and anti‐inflammatory role of free curcumin and curcumin‐loaded nanoparticles in neurotoxicity and other neurodegenerative diseases (Singh et al., 2017), (Barbara et al., 2017; Flora et al., 2013; Huang et al., 2017; Maithilikarpagaselvi et al., 2016; Motterlini et al., 2000; Sandhir et al., 2014; Tiwari et al., 2013; Yadav et al., 2012).

The nervous system among other biological systems is the most susceptible to lead toxic insults (Mahmoud & Sayed, 2016). The mechanism of lead intoxication in the brain results from its efficient ability to cross the blood–brain barrier and initiate various pathological alterations to membrane‐bound enzymes responsible for maintaining redox homeostasis, thus, causing oxidative stress which consequently leads to cell damage (Flora et al., 2012). Neuronal degeneration has been linked to signs of heavy metal‐induced neuronal death (Bhattacharjee et al., 2018). Toluidine blue stain is an important special stain for the brain tissue specifically Nissl bodies, nerve cells, and glia (Sridharan & Shankar, 2012). The basic thiazine metachromatic dye has a high affinity for acidic tissue components (nucleic acid blue and polysaccharides purple). It improves and enhances the sharpness of histological images (Sridharan & Shankar, 2012). In this study, the histological and histochemical analyses of the cerebral cortex from lead‐induced rats revealed marked cerebral damage with prominent alterations in the neuronal cells and necrosis, which resulted in a significant increase in the number of nonviable cells observed. Findings in this study are in agreements with the work of Sidhu and Nehru (2004), Mahmoud and Sayed (2016), and Lazarus et al. (2018), who reported extensive histoarchitectural distortions on the cerebral cortex of mice and rats characterized by cellular shrinkages, vacuolations, neuronal cell damages, hyperchromatic cells, and pyknosis due to lead intoxication. Furthermore, the present study demonstrated multiple pathological lesions on the cerebellum of lead‐induced rats. The histochemical and histological results revealed an area of degenerations with hyperchromatic Purkinje cells, scattered glial cells, and vacuolar spaces. The quantitative histological methods also revealed a significant increase in the number of nonviable Purkinje cells. These findings are in accordance with preceding literatures that documented the sensitivity of the Purkinje cells to lead exposure, and further reported Purkinje cells damage and perineural spaces in the cerebellum of lead‐exposed rats (Husain, 2015; Saleh & Meligy, 2018; Sidhu & Nehru, 2004; Yusuf et al., 2017). Brain tissue injury may be reversible (healing) or irreversible (permanent cell death) (Chongtham & Agrawal, 2016). However, this study clearly demonstrated the potential benefits of Cur‐CSCaCO_3_NP against lead‐induced toxicity. Correspondingly, administration with Cur‐CSCaCO_3_NP regardless of the dose has shown a better amelioration pattern when compared to the free curcumin treatment owing to its antioxidant property. In the same manner, curcumin‐loaded lipid nanoparticles revealed a higher ameliorative effect than free curcumin in reversing the pathological alterations induced by aluminum chloride in mice brain section (Kakkar & Kaur, 2011). In addition, several studies on curcumin and curcumin‐loaded nanoparticles have reported the combined antioxidant and anti‐inflammatory roles of free curcumin and nanoencapsulated curcumin using different nanoparticles in neurodegenerative diseases (Huang et al., 2017; Karimfar et al., 2014; Li et al., 2016; Noorafshan et al., 2013; Partadiredja et al., 2013; Sandhir et al., 2014).

## 6 CONCLUSION

**FIGURE 20:**
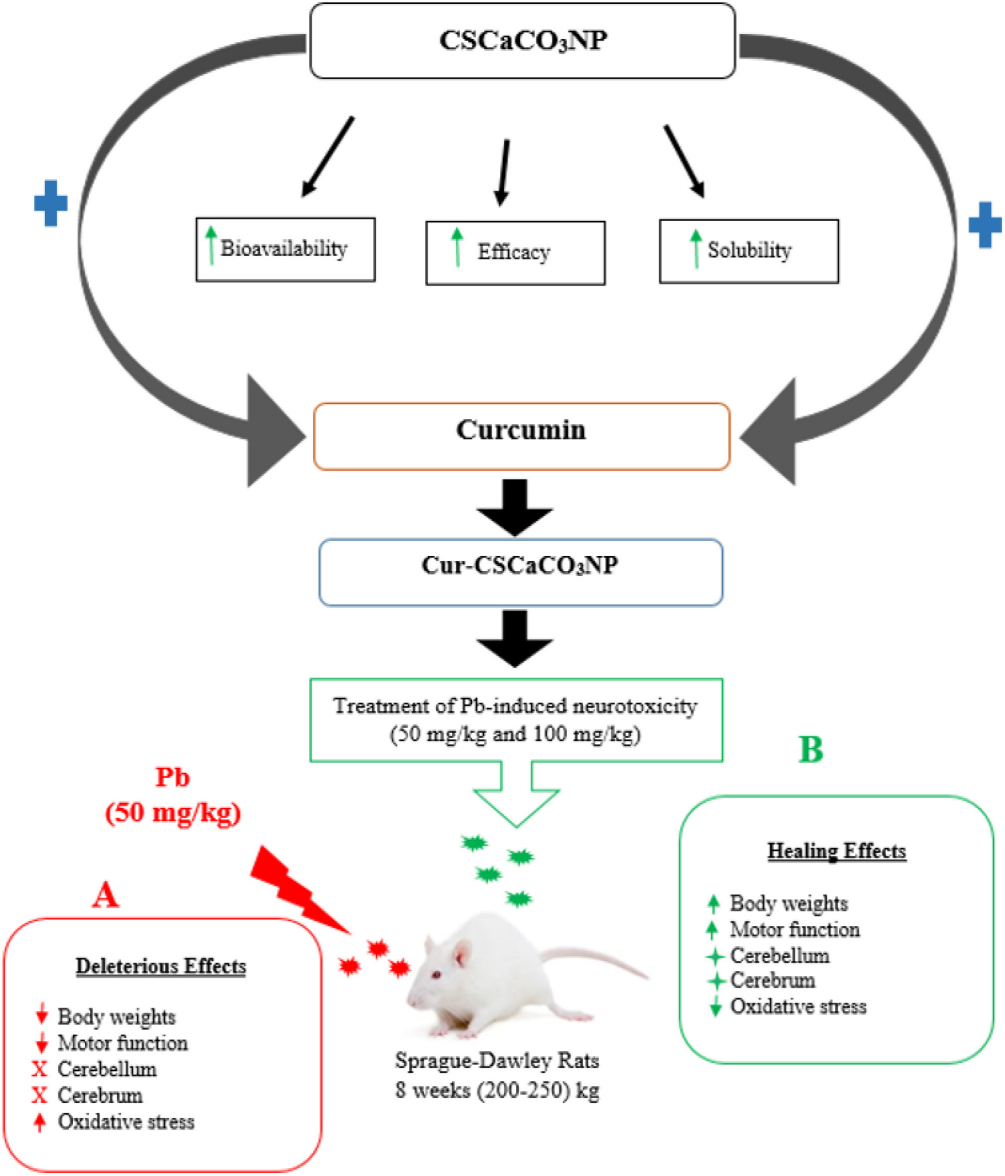
Proposed mechanism of lead‐induced neurotoxicity and the ameliorative effects of cur‐CSCaCO_3_NP. Cockle shell‐derived calcium carbonate nanoparticles (CSCaCO_3_NP); curcumin‐loaded cockle shell‐derived calcium carbonate nanoparticles (Cur‐CSCaCO_3_NP).

This study has shown that lead induction in rats resulted in oxidative stress, decreased body weight, and deficit in motor functions. In addition, lead induction in rats for 4 weeks was enough to affect the normal histology and functions of the cerebrum and cerebellum, which cast some histological aberrations such as cell degeneration and necrosis. Treatment with Cur‐CSCaCO_3_NP, irrespective of the dose given, demonstrated a higher therapeutic effect than free curcumin treatment by suppression of neuronal injury caused by lead through enhanced reduction in oxidative stress and reversed neurodegeneration as proven by histological and histochemical analysis. This new formulation of Cur‐CSCaCO_3_NP did not only demonstrate the enhanced improvement of antioxidant effect but also potentially attenuated motor function deficit (Figure 20). These findings demonstrated that Cur‐CSCaCO_3_NP could effectively decrease the damage associated with oxidative stress. Thus, CSCaCO_3_NP can be considered a brain‐targeted delivery system. Cur‐CSCaCO_3_NP is a novel approach for the treatment of lead‐induced neurotoxicity and a potential therapeutic for neurodegenerative diseases. Although this study investigated the antioxidant effects of Cur‐CSCaCO_3_NP on lead‐induced neurotoxicity in rats via assessments of SOD activities and MDA levels, further studies are warranted to decipher the antioxidant effect of Cur‐CSCaCO_3_NP on other antioxidant enzymes such as GSH, catalase, glutathione reductase, and peroxidase. Further studies on the mechanism of how Cur‐CSCaCO_3_NP crosses the blood‐brain barrier to execute its therapeutic effects should be studied. This might provide an additional clue on the mechanism behind the antioxidant effect of Cur‐CSCaCO_3_NP.

## CONFLICTS OF INTEREST

The authors report no conflicts of interest in this work. The authors alone are responsible for the content and writing of this article.

## Supporting information


**Figure S1** **–S2**

## Data Availability

1. The data that support the findings of this study are openly available in the bibliography section.2. The data that support the findings of this study are available in URL/DOI under the bibliography section.
