# Supplementary material for: Curcumin‐loaded cockle shell‐derived calcium carbonate nanoparticles ameliorates lead‐induced neurotoxicity in rats via attenuation of oxidative stress
Source: Food Sci Nutr. 2022 Oct 30;11(5):2211–31. doi: 10.1002/fsn3.3096 (PMC10171497; doi:10.1002/fsn3.3096)
Supplement: Supplementary file 1 — Figure S1 –S2 [file FSN3-11-2211-s001.docx]

**List of Appendix**

Appendix A


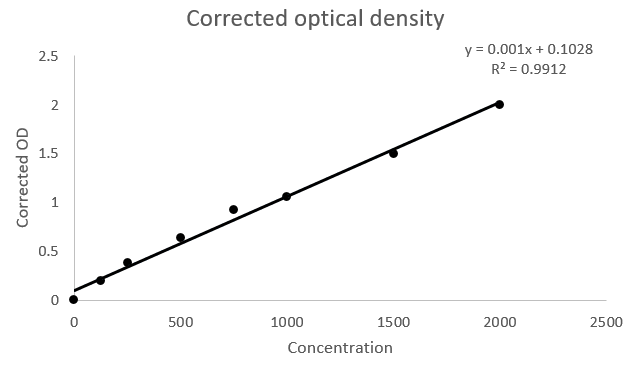


**Fig. A. 1.** Linearity curve for standard protein estimation for BCA assay.

Appendix B

ELISA Analysis


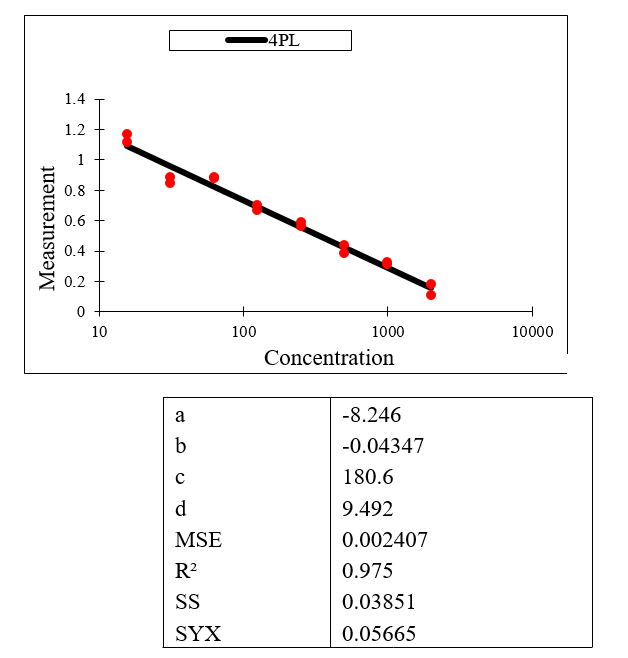


**Fig. A. 2.** Standard calibration curve for MDA assay
